# Bioassay-Guided Isolation of Nigracin, Responsible for the Tissue Repair Properties of *Drypetes Klainei* Stem Bark

**DOI:** 10.3389/fphar.2019.01541

**Published:** 2020-01-23

**Authors:** Gianluca Sferrazza, Marco Corti, Federica Andreola, Daniela Giovannini, Giuseppe Nicotera, Manuela Zonfrillo, Massimo Serra, Sara Tengattini, Enrica Calleri, Gloria Brusotti, Pasquale Pierimarchi, Annalucia Serafino

**Affiliations:** ^1^Institute of Translational Pharmacology—National Research Council of Italy, Rome, Italy; ^2^Department of Drug Sciences, University of Pavia, Pavia, Italy

**Keywords:** *Drypetes klainei* Pierre ex Pax, bark extract, wound healing, nigracin, bioassay-guided isolation

## Abstract

*Drypetes klainei* Pierre ex Pax is used in Cameroon by Baka people in the wound healing process and for the treatment of burns. In a previous paper we demonstrated the ability of both water (WE) and defatted methanol (DME) extracts to accelerate scratch wound closure in fibroblast cultures, thus validating the traditional use of *D. klainey* stem bark in the treatment of skin lesions. In this work we carried out a bioassay-guided fractionation of the most active DME, which exhibited *in vitro* efficacy in accelerating wound healing process, in order to isolate and identify the compound/s responsible for the assessed biological activity. HPLC was used for the metabolite profiling of DME and fractions (analytical) and for the isolation of the bioactive compound (semi-preparative). MS analyses and NMR spectroscopy were used for identifying the isolated compound. The abilities of treatments in accelerating wound healing were studied on murine fibroblasts in terms of cell viability and cell migration (scratch wound-healing assay). The results obtained allowed to unambiguously identify the isolated bioactive compound as nigracin, a known phenolic glycoside firstly isolated and characterized from bark and leaves of *Populus nigra* in 1967. However, this is the first time that nigracin is identified in the *Drypetes* genus and that a wound healing activity is demonstrated for this molecule. Specifically, we demonstrated that nigracin significantly stimulates fibroblast growth and improves cell motility and wound closure of fibroblast monolayer in a dose-dependent manner, without any toxicity at the concentrations tested, and is still active at very low doses. This makes the molecule particularly attractive as a possible candidate for developing new therapeutic options for wound care.

## Introduction

The traditional medicinal uses of 19 species of the genus *Drypetes*, distributed in Africa and Asia, and the isolation of about 150 compounds have been reported in a rather recent review (Wansi et al., 2016). Despite the huge number of the potential pharmacological properties attributed to this genus, in this paper the validation of the traditional uses is reported only for five species, *D. chevalieri, D. gerrardii, D. gosswelieri, D. natalensis, D. roxburghii*.

However, in a previous paper (Brusotti et al., 2015) we described the validation of the traditional use of *Drypetes klainei*, known among Baka people in Cameroon as a remedy in the wound healing process and in the treatment of burns.

In order to complete the research on this plant, a bioassay-guided fractionation of the most active defatted methanol extract (DME), which exhibited *in vitro* efficacy in accelerating wound healing process (Brusotti et al., 2015), was carried out, aiming to isolate and identify the compound/s responsible for the assessed biological activity.

**Figure 1:**
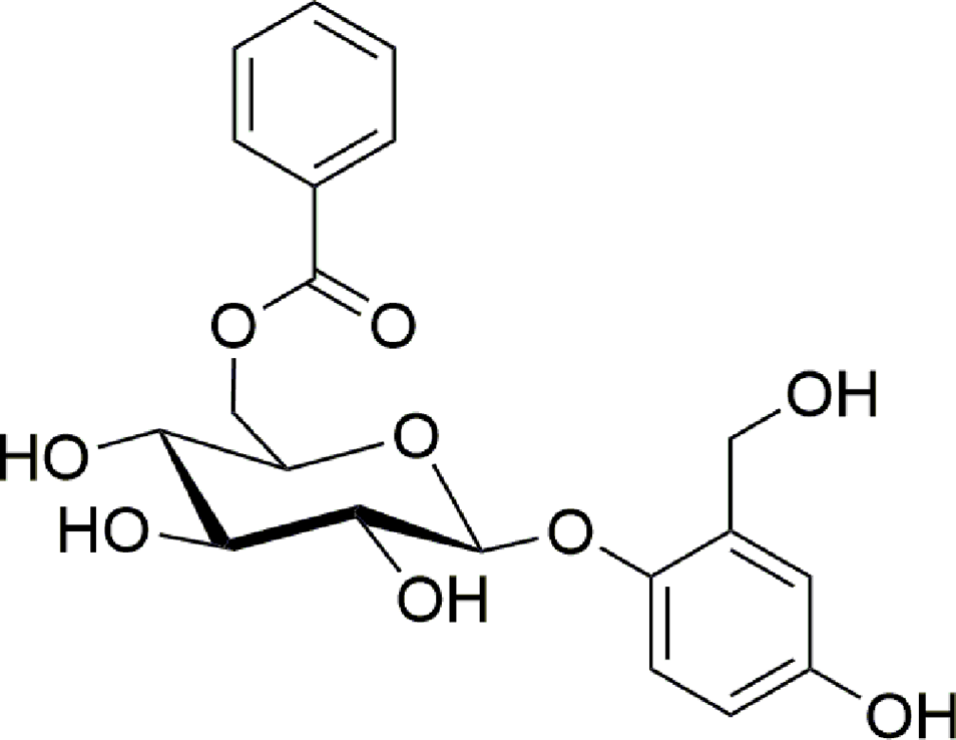
Molecular structure of nigracin.

Particularly, we described the purification procedures and the biological assays that allowed to isolate a pure compound responsible for the wound healing activity and the analytical techniques needed to identify it as the known compound nigracin (**Figure 1**).

Nigracin was first isolated and characterized from bark and leaves of *Populus nigra* in 1967 (Thieme and Benecke, 1967); three years later (Thieme, 1970) the same author reported that nigracin, xylosmoside, isolated in 1954 from *Xylosma apactis* (Fukui, 1954), and poliothirsoside, isolated in 1969 from *Poliothirsis sinensis* (Plouvier, 1969) were the same compound. More recently nigracin was found in bark and twigs of *Itoa orientalis* (Chai et al., 2007), in leaves and twigs of *Flacourtia indica* (Sashidhara et al., 2013), in stems of *Homalium ceylanicum* (Liu et al., 2013) but, as far as we know, nigracin was never found before in the genus *Drypetes*.

Several biological properties have been attributed to this phenolic glycoside: antimalarial activity (Kaou et al., 2010; Sashidhara et al., 2013), antidiabetic potential (Sashidhara et al., 2014), anti-inflammatory activity against COX-2 (Chai et al., 2008).

In this work, we reported the isolation of nigracin from *Drypetes klainei* and its activity in the wound healing process, both described here for the first time.

## Materials and Methods

### Chemicals and Reagents

Acetonitrile, methanol, trifluoroacetic acid (all of HPLC grade) were purchased from Sigma-Aldrich Co. (USA). Sterile deionized water was produced by a water purification system (Milli-Q Reagent Water System, MA, USA).

### Preparation of Plant Extracts

The stem bark of *Drypetes klainei* was collected in Cameroon in July 2011 in the camps of Abing and identified and stored at the University of Pavia as previously described (Brusotti et al., 2015). The plant was identified at the National Herbarium of Yaoundé by the Cameroonian botanist Mr. Victor Nana, and was provided according to a Material Transfer Agreement. A voucher specimen (no. BWPV 10) has been deposited at the Department of Drug Sciences of the University of Pavia, which has a Research Collaboration Agreement with the University of Yaoundé. The defatted methanol extract (DME) was obtained following the extraction procedure previously described (Brusotti et al., 2015).

### HPLC-DAD

HPLC was used for the metabolite profiling of DME and fractions (analytical) and for the isolation of the bioactive compound (semi-preparative). Chromatographic experiments were performed with an Agilent 1100 liquid instrument (Palo Alto, CA, USA) equipped with an Agilent 1100 variable-wavelength detector. The system was connected to an HPLC ChemStation. (RevisionA.04.01). Analytical RP-HPLC analyses were carried out with a Agilent Technologies LiChrospher 100 RP-18 column (5 µm) 250×4 mm, using a step gradient system of 0.04% TFA in H_2_O (solvent A) and MeOH (solvent B), as follow: 5% to 30% of B in 12 min, 30% to 41% of B in 25 min, 41% to 60% of B in 40 min, 60% to 100% of B in 10 min; flow rate: 0.7 ml/min. 10 mg of DME or fractions were dissolved in 1 ml of 90/10 H_2_O/MeOH. The obtained suspensions were then sonicated for 5 min and centrifugated for 8 min at 4500 RPM. Therefore, the supernatant was injected using a sample loop of 20 μl. The UV absorbance was measured at 225 nm.

Semi-preparative RP-HPLC was carried out with Atlantis® Prep T3 5 µm 10×150 mm column, using a step gradient system of 0.04% TFA in H_2_O (solvent A) and CH_3_CN (solvent B), as follow: 20% to 26 % of B in 12 min, 26 % to 100 % of B in 3 min, 100 % of B for 5 min, from 100 % to 20 % of B in 1 min. Flow rate: 3 ml/min. 5 mg of DME were dissolved in 5 ml of 90/10 H_2_O/MeOH. The obtained suspensions were then sonicated for 5 min and centrifuged for 8 min at 4500 RPM. Therefore, the supernatant was injected using a sample loop of 1 ml. The UV absorbance was measured at 225 nm.

### MS Analyses

MS data were obtained using a Linear Trap Quadrupole (LTQ) mass spectrometer equipped with an electrospray ionization (ESI) source (Thermo Finnigan, San Jose, CA, USA). The system was controlled by Xcalibur software 1.4 (Thermo Finnigan). The sample was dissolved in MeOH and directly introduced into the mass spectrometer by continuous infusion at a rate of 10 µL/min. Full scan MS experiments were carried out under the following instrumental conditions: positive ion mode; mass range, 150–1000 m/z; source voltage, 4.6 kV; capillary voltage, 49 V; sheat gas, 6 (arbitrary units); auxiliary gas, 1 (arbitrary units); capillary temperature, 250 °C; tube lens voltage, 106 V. MS/MS spectra were obtained by collision-induced dissociation (CID) with a normalized collision energy of 35.0.

### NMR Spectroscopy

Nuclear magnetic resonance spectra were acquired using a Bruker Avance 400 MHz spectrometer equipped with Bruker’s TopSpin 1.3 software package. The abbreviations s, d, dd, and m stand for the resonance multiplicities singlet, doublet, doublet of doublet, and multiplet, respectively. In the peak listing of ^13^C spectra abbreviations s and t refer to zero and two protons attached to the carbons, as determined by DEPT-135 experiments. Sample temperatures were controlled with the variable-temperature unit of the instrument.

### Bioassay-Guided Fractionation and Isolation of Nigracin

The DME was further purified by flash chromatography, applying the procedure previously described by Still et al., (1978). 8 cm of a glass column (5 cm diameter) were filled with Silica LiChroprep® RP-18 (25-40 µm, MERCK). 400 mg of DME were suspended in 10 ml of H_2_O/MeOH 80/20, sonicated for 5 min and then centrifuged for 8 min at 4500 RPM. The obtained supernatant was charged on the column. The first solvent system (H_2_O/MeOH 70/30 + 0.04% TFA) was added to the column and the elution started at the flow rate of 25 ml/min; 300 ml were necessary to complete the elution of fraction 1 (Fr1). Further 300 ml of the next solvent system (H_2_O/MeOH 50/50 + 0.04% TFA) were necessary for the elution of fraction 2 (Fr2); further 300 ml (100% MeOH + 0.04% TFA) for the elution of fraction 3 (Fr3). The column was finally washed with 300 ml of 100% MeOH. After evaporation of the solvent *under vacuum*, Fr1 (204 mg), Fr2 (98 mg) and Fr3 (57 mg) were subjected to biological analyses.

The most active fraction (Fr2) was further purified by flash chromatography. 8 cm of a glass column (5 cm diameter) were filled with Silica LiChroprep^®^ RP-18 (25-40 µm, MERCK). 300 mg of Fr2 were suspended in 10 ml of H_2_O/MeOH 90/10, sonicated for 5 min and then centrifuged for 8 min at 4500 RPM. The obtained supernatant was charged on the column. The first solvent system (H_2_O/MeOH 90/10 + 0.04% TFA) was added to the column and the elution started at the flow rate of 25 ml/min; 300 ml were necessary to complete the elution of fraction 1 (Fr2subA). Further 1200 ml of the next solvent system (H_2_O/MeOH 70/30 + 0.04% TFA) were necessary for the elution of fraction 2 (Fr2subB, 600 ml) and for fraction 3 (Fr2subC, 600 ml). Fraction 4 (Fr2subD) and fraction 5 (Fr2subE) were then eluted using 300 ml of H_2_O/MeOH 50/50 + 0.04% TFA, 300 ml respectively. The column was finally washed with 300 ml of 100 % MeOH. After evaporation of the solvent *under vacuum*, Fr2subA (91.5 mg), Fr2subB (16.5 mg), Fr2subC (29 mg), Fr2subD (31 mg), Fr2subE (36 mg) were subjected to biological analyses.

Based on the biological activities highlighting the presence of the bioactive compound in the almost pure fraction Fr2subE, a semi-preparative RP-HPLC was applied directly to the DME, allowing the isolation of nigracin, whose chemical characterization was achieved by NMR and MS analyses, as described above.

### Cell Culture Used and Treatments

The mouse embryonic fibroblast cell line NIH 3T3 (3T3; ATCC, Manassas, VA, USA), was grown in Dulbecco’s Minimal Essential Medium (DMEM), supplemented with 10 % heat-inactivated Fetal Bovine Serum (FBS), L-glutamine (2 mM), penicillin (100 IU/ml) and streptomycin (100 µg/ml). All media and supplements were obtained from Hyclone (Logan, UT, USA). Cells were maintained at 37 °C, in a humidified atmosphere of 5 % CO_2_, and passaged after being detached from culture flasks with 0.05 % trypsin and 0.002 % EDTA solution.

Exponentially growing cells were seeded at a density of 4 × 10^4^/cm^2^ and were maintained in culture for 24 h before treatments. Cells were treated with increasing doses of fractions or purified nigracin, ranging from 0.015 to 50 µg/ml, and maintained in culture up to 24 h before to be analyzed. The effects of nigracin on cell viability and wound scratch closure were also compared with those of hyaluronic acid (HA; MW 500-700 kDa. Fidia Farmaceutici S.p.A., Italy), used as positive control of wound healing stimulation. In all experiments, untreated controls consisted of cells cultured in basal medium.

### Cell Viability and Cell Cycle Analyses

Cell viability was determined after 24 h of treatment based on the Trypan blue dye exclusion method. Results were reported, as a mean of three independent experiments.

The effect of the treatments on cell cycle was evaluated by cytofluorimetric analysis of DNA content after propidium iodide (PI) staining, using the FACSCalibur flow cytometer (Becton Dickinson, Franklin Lakes, NJ, USA).

### Scratch Wound-Healing Assay

3T3 fibroblasts were seeded in 6-well plates (8 × 10^5^ cells/well) and grown until reached a confluence of 90-95%, in the proper culture conditions described above. The scratch wound assay was performed as previously described (Rodriguez et al., 2005; Brusotti et al., 2015). The cultures subjected to scratch wound was exposed to increasing doses of fractions, sub-fractions and isolated compound nigracin (ranging from 0.3 to 50 μg/ml, from 0.3 to 6 μg/ml, and from 0.015 to 12 μg/ml, respectively) for 24 h at 37 °C in a humidified atmosphere of 5 % CO_2_. Fibroblasts grown in basal medium were used as untreated control. Scratch wound closure was analyzed, as previously described (Brusotti et al., 2015), in two modalities: i) the static imaging modality, performed under the phase-contrast microscope Motic AE31 (Motic, Milan, Italy) equipped with a digital CCD camera, by acquiring two digital images of the wound at time 0 (T0), and 24 h (T24h) after the addition of treatments to culture medium; ii) by time-lapse imaging in bright field, carried out using the July Br Cell Movie Analyser (NanoEnTek, Seoul, Korea), used for analyzing the wound healing dynamic. The static imaging modality was used in preliminary dose-response experiments, to select the most effective dose/s to be used in the time-lapse experiments. The closure of the scratch was quantified by measuring the difference between the wound width at T0 and T24, using the ImageJ processing software [http://rsbweb.nih.gov/ij/], and the scratch closure rate (SCR) was calculated, as recently described by us and others (Brusotti et al., 2015; Felice et al., 2015), using the following formula:

$$SCR=\left[\frac{\left(At0-At24\right)}{At0}\right]\times 100$$

where *At0* = scratch area at time 0; *At24* = scratch area at time 24 h. Results were reported as the mean of three independent experiments ± SD.

The doses of fractions or isolated compound, selected from the preliminary dose-response experiments, were then tested for their ability to accelerate wound healing *in vitro*, by time-lapse imaging in bright field, as previously described (Brusotti et al., 2015). 3T3 cells were monitored for 24 h of culture in absence and in presence of treatments, by acquiring 1 frame/5 min. During the acquisition, the wound healing rate was automatically calculated by the July Br Cell Movie Analyser and recorded as percentage of confluence in function of time. From the quantitative data of the wound healing dynamic, we calculated, as previously described (Brusotti et al., 2015), the wound healing index (WH) using the formula:

$$WH\;index\;=\;(CoeffLT\;\times \;Final\;\%\;Confluence)\;+\;\frac{1}{y\;Intercept}$$

where *CoeffLT* = slope coefficient of linear trend-line equation (directly correlated to the speed of wound closure); *y* Intercept = the point where the trend-line crosses the *y* axis (inversely correlated to the time at which the first migratory movement is recorded and, therefore, to the promptness of the effect). The linear trend-line equation for each curve was obtained directly by the Excell software. On frames at T0 and T24h from the time-lapse acquisitions, the SCRs were also obtained as described above.

### Statistical Analysis

Statistical analyses were conducted using the two-tailed Student’s t test and a *p* value threshold of ≤ 0.05. All data were presented as mean ± SD.

## Results

### Identification of the Fraction/S, Obtained from Defatted Methanol (DME) Extract, Possessing Wound Healing Activity

**Figure 2:**
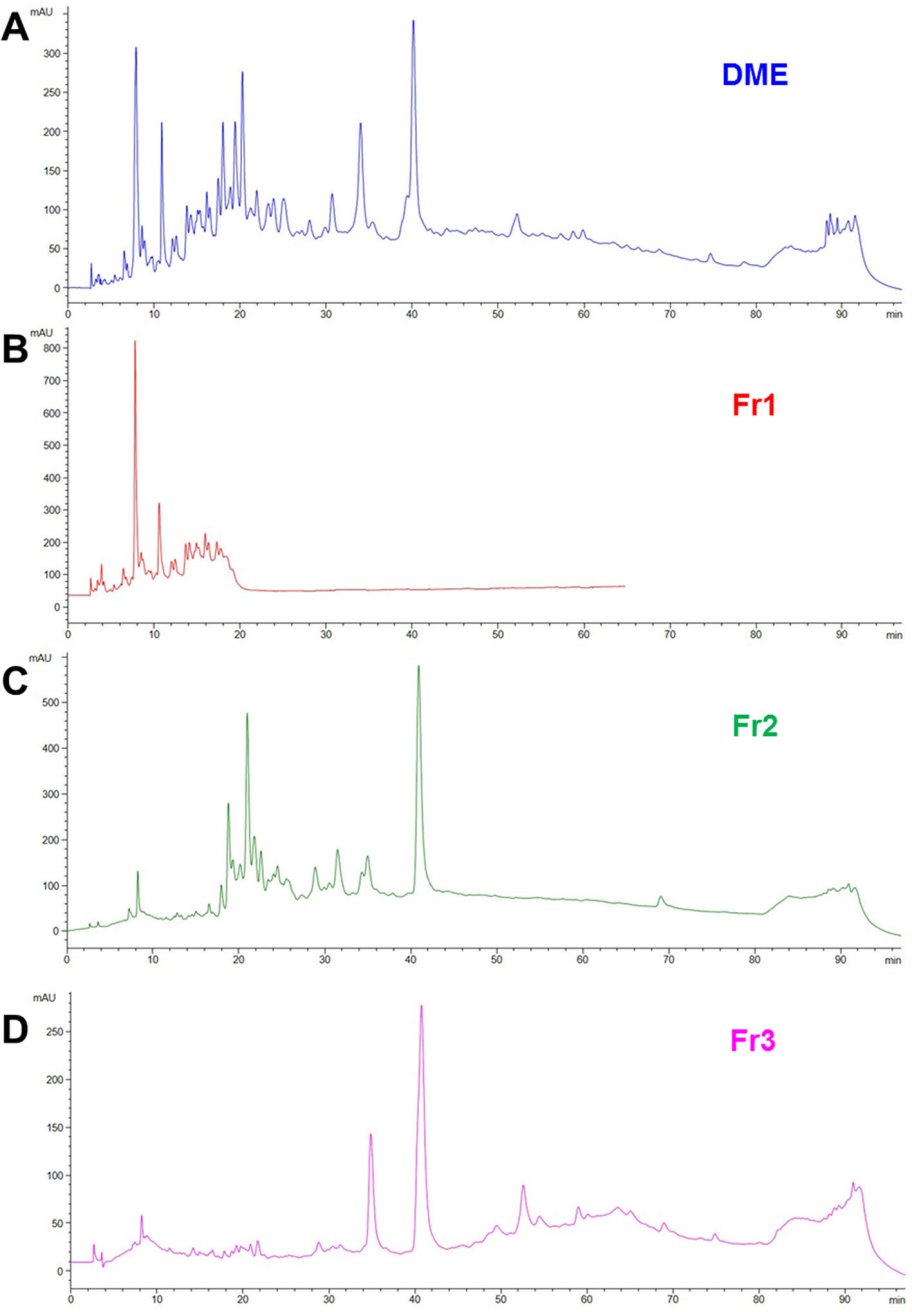
RP-HPLC-DAD fingerprinting of DME extract **(A)**, Fr1 **(B)**, Fr2 **(C)** and Fr3 **(D)** fractions. Chromatograms obtained by the injection of 20 μl of 10 mg/ml samples using a step gradient system of 0.04% TFA in H_2_O (solvent A) and MeOH (solvent B), as follow: 5% to 30% of B in 12 min, 30% to 41% of B in 25 min, 41% to 60% of B in 40 min, 60% to 100% of B in 10 min; flow rate: 0.7 ml/min. Detection at 225 nm.

We previously demonstrated that both water (WE) and defatted methanol (DME) extracts from *Drypetes klainei* stem bark significantly improve the wound healing process *in vitro*, compared to untreated controls, and that the DME was active at lower concentrations, compared to the WE. Based on these results, DME was purified by flash chromatography giving rise to three fractions, Fr1, Fr2 and Fr3 showing the chromatographic profiles reported in (**Figure 2**), in comparison with that of the whole DME.

**Figure 3:**
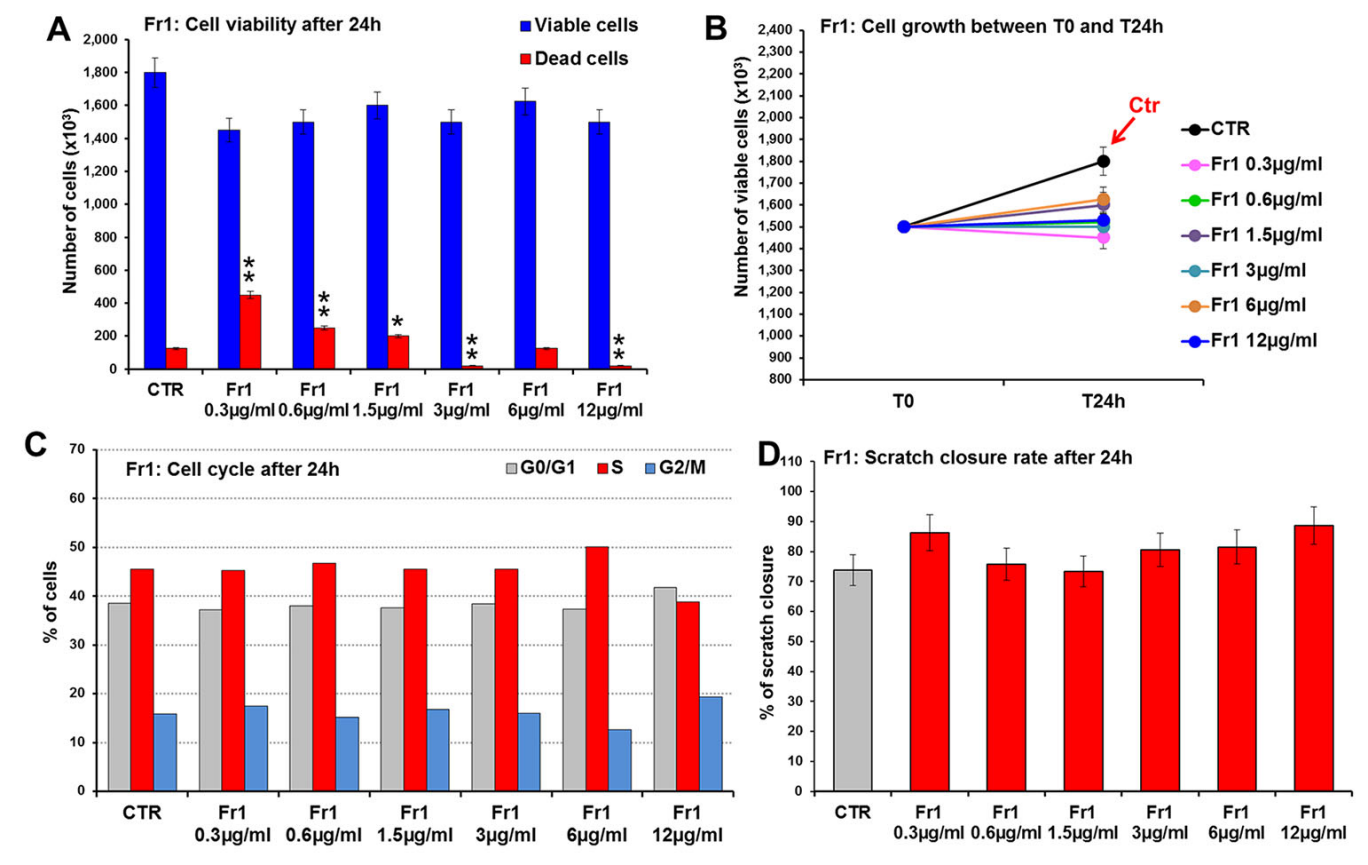
Effect of Fr1 from DME extract on viability, growth and cell cycle of murine fibroblasts and on scratch wound closure in the cell monolayer. 3T3 cells were treated with 0.3, 0.6, 1.5, 3, 6, 12 µg/ml of Fr1, and analyzed after 24 h. **(A, B)** Cell viability **(A)** and growth **(B)**, evaluated by Trypan blue dye exclusion method, in untreated controls and in Fr1 treated cells. **(C)** Cell cycle analysis by flow cytometry performed on 3T3 cells after 24 h of treatment, and in the untreated controls. **(D)** Bar graph of scratch closure rates (SCR) at T24 from static imaging modality, calculated as described in the method section. Significance *vs* untreated control (CTR): *p < 0.05; **p < 0.01; the mean ± SD; n = 3.

Fr1, Fr2, and Fr3 were then tested for their ability to accelerate wound healing process in murine fibroblasts, in terms of cell viability and migration (scratch wound assay). To this purpose, 3T3 cells were treated for 24 h with increasing concentrations (ranging from 0.3 to 50 µg/ml) of each fraction and firstly analyzed for cell viability and growth and for cell cycle. Compared to the untreated control, the Fr1 did not produce any significant effect of cell growth and on the number of viable cells (**Figures 3A, B**), nor on cell cycle (**Figure 3C**), while partial cytotoxicity at the lower concentration used has been recorded (**Figure 3A**). Moreover, Fr1 was not able to significantly accelerate wound healing process compared with the untreated control (**Figure 3D** and **Supplementary Figure 1**).

**Figure 4:**
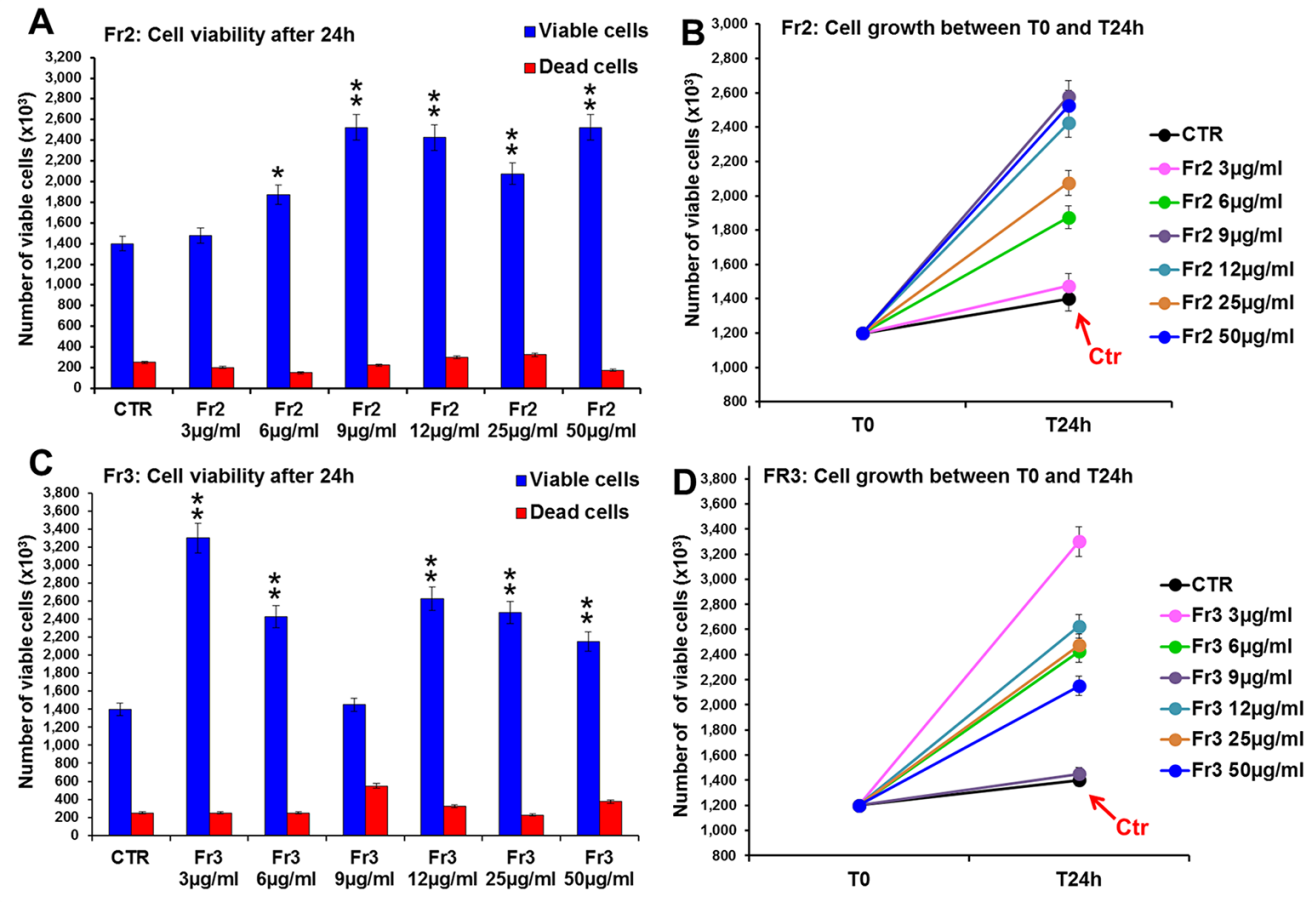
Comparative analysis of the effect of increasing concentrations of Fr2 and Fr3 fractions on 3T3 cell viability and growth. Cell viability **(A, C)** and growth **(B, D)**, were evaluated by Trypan blue dye exclusion method, in untreated controls and cells treated for 24 h with 3, 6, 9, 12, 50 µg/ml of Fr2 **(A, B)** and Fr3 **(C, D)**. Significance *vs* untreated control (CTR): *p < 0.05; **p < 0.01; the mean ± SD; n = 3.

Conversely, both Fr2 and Fr3 fractions significantly increased cell growth and viability (**Figure 4**), with absent or very low toxicity, but the Fr2 fraction functioned in a dose-dependent manner (**Figures 4A, B**) compared with the Fr3 fraction, whose effects were higher at the lower concentration and discontinuously decreased with the increment of the doses (**Figures 4C, D**).

Consistently with the higher growth rate recorded by the viability assay, the cytofluorimetric analysis of cell cycle performed after 24 h of treatment (**Supplementary Figure 2**) showed that both Fr2 and Fr3 fractions induced a moderate increment of the percentage of cells in S phase compared to the untreated control (up to 19 % and 14.8 % of increment for Fr2 and Fr3, respectively).

**Figure 5:**
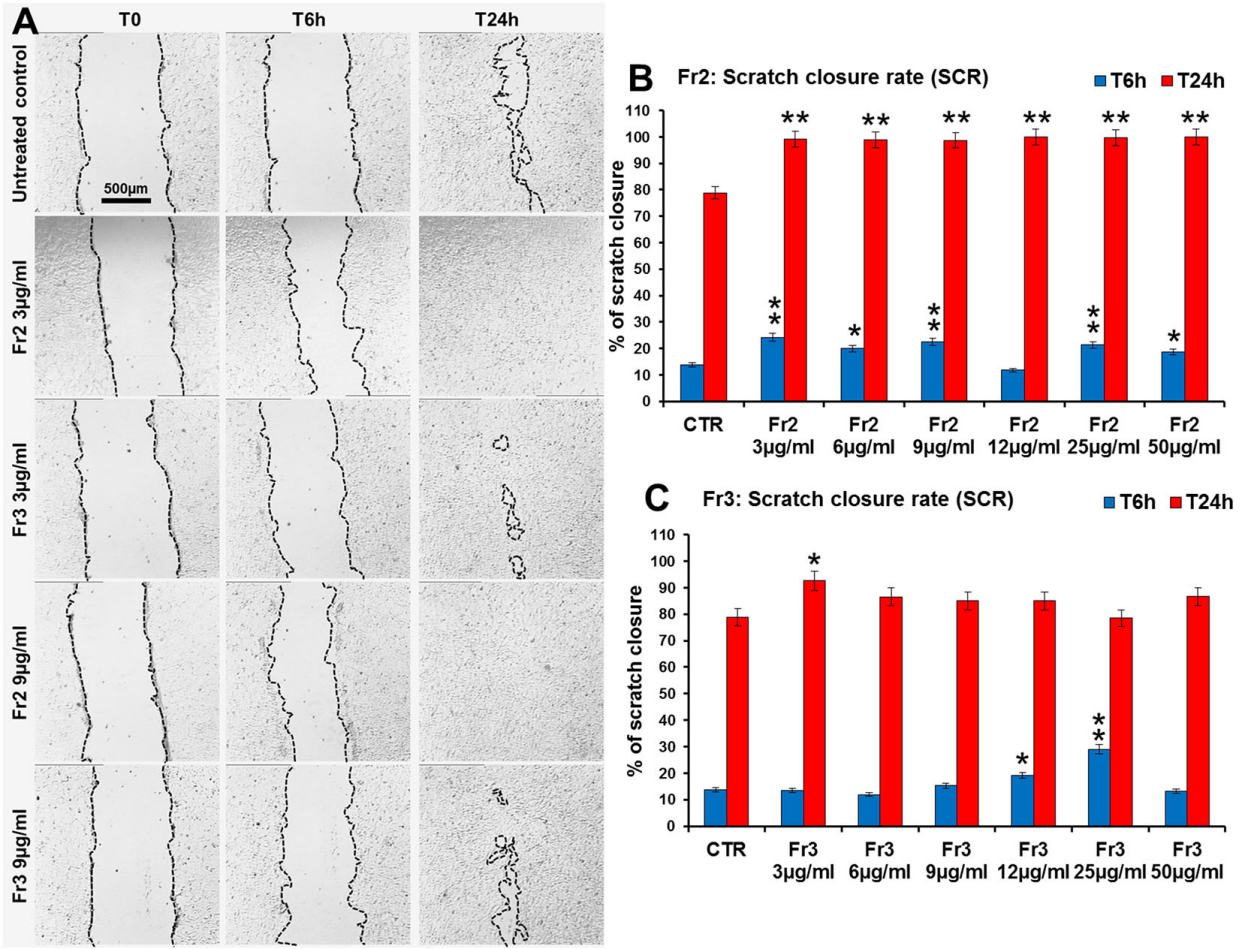
Comparative analysis of the effect of increasing concentrations of Fr2 and Fr3 fractions on scratch wound closure in 3T3 cell monolayer. 3T3 cells were treated with 3, 6, 9, 12, 25 and 50 µg/ml of Fr2 or Fr3 and wound closure was analyzed after 6 h (T6h) and 24 h (T24h) of treatment by static imaging modality. **(A)** Representative images, by phase contrast microscopy, of 3T3 cells before (T0) and after 6 h (T6h) and 24 h (T24h) of treatment with 3 and 9 µg/ml of Fr2 and Fr3 fractions, compared to the untreated control. Bar: 500 µm. **(B, C)** Bar graphs of scratch closure rates (SCR) at T6h and T24h, calculated as described in the method section. Significance *vs* untreated control (CTR): *p < 0.05; **p < 0.01; the mean ± SD; n = 3.

Moreover, the comparative analysis of the effect of increasing concentrations (3, 6, 9, 12, 25, 50 µg/ml) of Fr2 and Fr3 fractions on scratch wound closure examined at the times 0 (T0), 6 h (T6h) and 24 h (T24h) after wounding, showed that for the Fr3 fraction the scratch wound closure was significantly accelerated only by the lower concentration at T24h (1.18-fold *vs* control) and by the concentrations 12 and 25 µg/ml (1.4-fold and 2.1-fold *vs* control, respectively) at T6h (**Figures 5A, C** and **Supplementary Table 1**). Conversely, all the concentrations used for the Fr2 resulted significantly active, at both times analyzed, on the wound healing process (*p* ≤ 0.05), with an increment up to 1.78-fold (at T6h) and 1.27-fold (at T24h) *vs* the untreated control (**Figures 5A, B** and **Supplementary Table 1**), that led to a complete scratch closure after 24 h already at the lower dose used (3 µg/ml). Actually, the Fr2 fraction retained its wound healing activity also when the concentration was lowered up to 0.015 µg/ml (**Supplementary Figure 3**).

### Identification of the Active Sub-Fraction/s Obtained from the Fr2 Fraction

**Figure 6:**
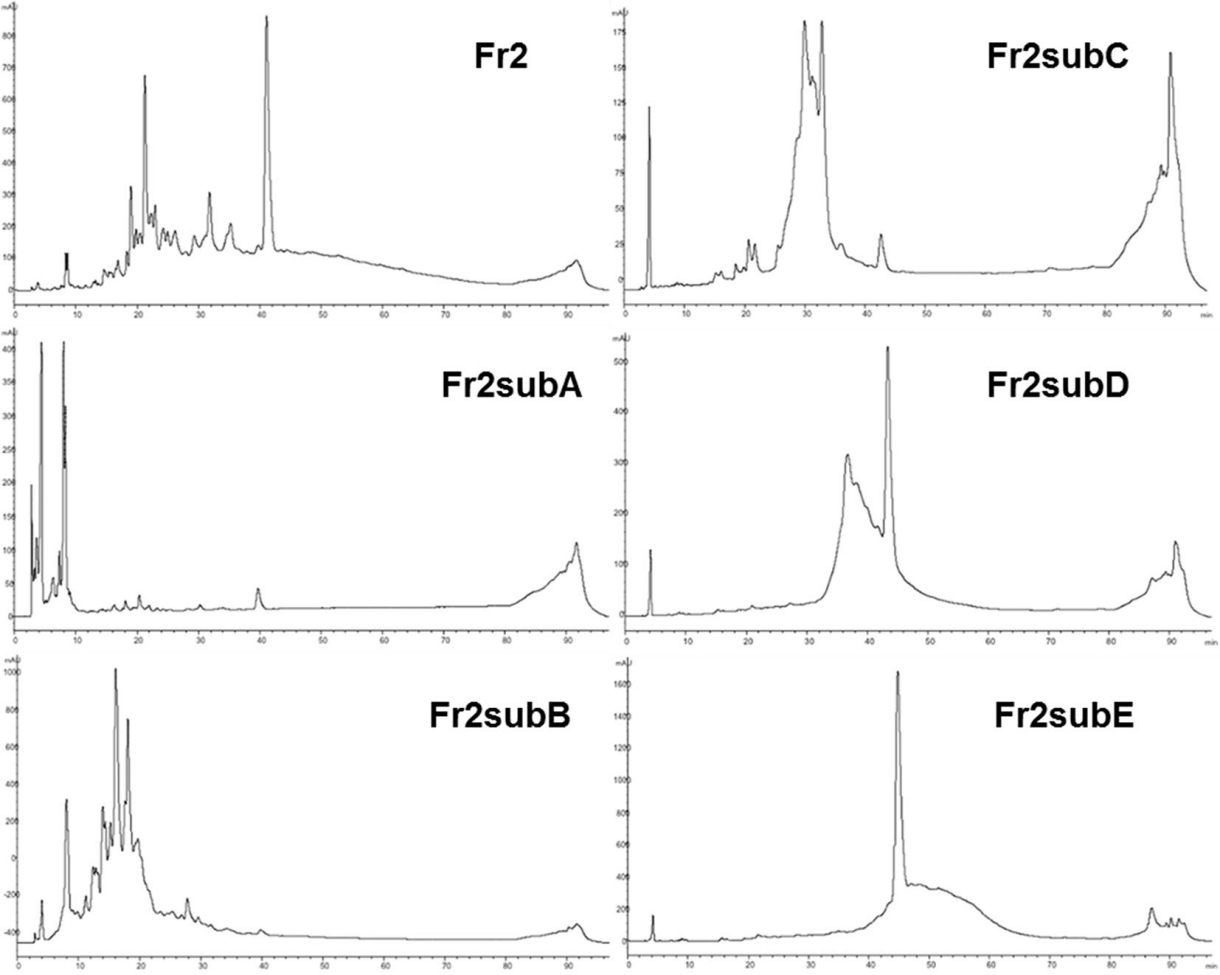
RP-HPLC-DAD fingerprinting of Fr2, Fr2subA, Fr2subB, Fr2subC, Fr2subD, Fr2subE. Chromatograms obtained by the injection of 20 μl of 10 mg/ml samples using a step gradient system of 0.04% TFA in H_2_O (solvent A) and MeOH (solvent B), as follow: 5% to 30% of B in 12 min, 30% to 41% of B in 25 min, 41% to 60% of B in 40 min, 60% to 100% of B in 10 min; flow rate: 0.7 ml/min. Detection at 225 nm.

The results obtained indicated that, even if both Fr2 and Fr3 fractions improved the wound healing process in terms of cell viability and migration, Fr2 was more potent and acted in a dose-dependent manner. Therefore, the most active Fr2 was further purified by flash chromatography affording five sub-fractions, Fr2subA, Fr2subB, Fr2subC, Fr2subD, Fr2subE. **Figure 6** shows the chromatographic profiles of the sub-fractions in comparison with those of the native Fr2.

The five sub-fractions were then subjected to the bioassays (cell viability and growth, cell cycle, scratch wound-healing) for testing their activity.

**Figure 7:**
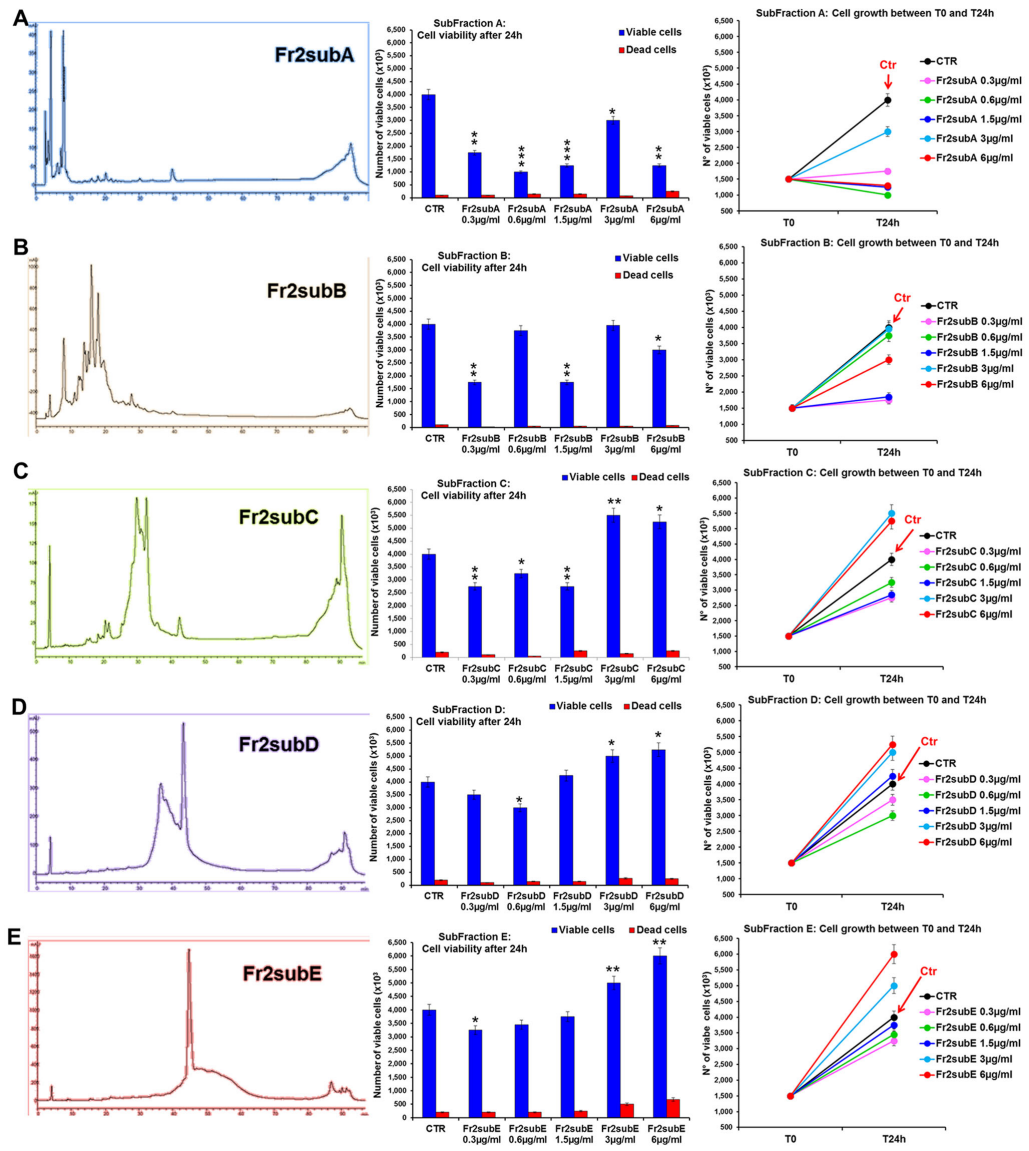
Comparative analysis of the effect of increasing concentrations of Fr2 sub-fractions A, B, C, D and E on 3T3 cell viability and growth. Cell viability (*middle panels*) and growth (*right panels*), were evaluated by Trypan blue dye exclusion method, in untreated controls and cells treated for 24 h with 0.3, 0.6, 1.5, 3, 6 µg/ml of Fr2subA **(A)**, Fr2subB **(B)**, Fr2subC **(C)**, Fr2subD **(D)** and Fr2subE **(E)**. In the *left panels*, the HPLC profiles of each sub-fraction were also reported. Significance *vs* untreated control (CTR): *p < 0.05; **p < 0.01; ***p < 0.001; the mean ± SD; n = 3.

The comparative analysis of the effect of increasing concentrations (0.3, 0.6, 1.5, 3, 6 µg/ml) of Fr2sub-fractions A, B, C, D and E on cell viability and growth (**Figure 7**) showed that both Fr2subD and Fr2subE significantly increased cell growth and viability in a dose-dependent manner, with a low toxicity (with a maximum of 10.11% dead cells for the sub-fraction E at the higher concentration used; **Supplementary Table 2**), but the effect was higher and more significant for the Fr2subE (**Figure 7E**). Conversely, the sub-fractions A, B and C, in general decreased cells growth (**Figures 7A, B, C**): in particular, Fr2subA negatively affected cells growth and viability (with a percentage of dead cells up to about 17%; **Supplementary Table 2**), Fr2subB seemed to exert a cytostatic rather than cytotoxic effect, having a very low cytotoxicity but inhibiting cell growth, and Fr2subC decreased or increased cell growth at the lower or at the higher concentrations used, respectively (**Figure 7C**).

Cell cycle analysis performed after 24 h of treatment (**Supplementary Figure 4**) confirmed that the Fr2subE was the unique fraction that induced a moderate but significant increment of the percentage of cells in S phase compared to the untreated control (**Supplementary Figure 4E**), while Fr2subD slightly increased the S phase only at the higher concentrations (**Supplementary Figure 4D**). Conversely, sub-fractions A, B, and C decreased the S phase and positively affected the percentage of cells in G0/G1 or in G2 phases (**Supplementary Figures 4A**–**C**).

**Figure 8:**
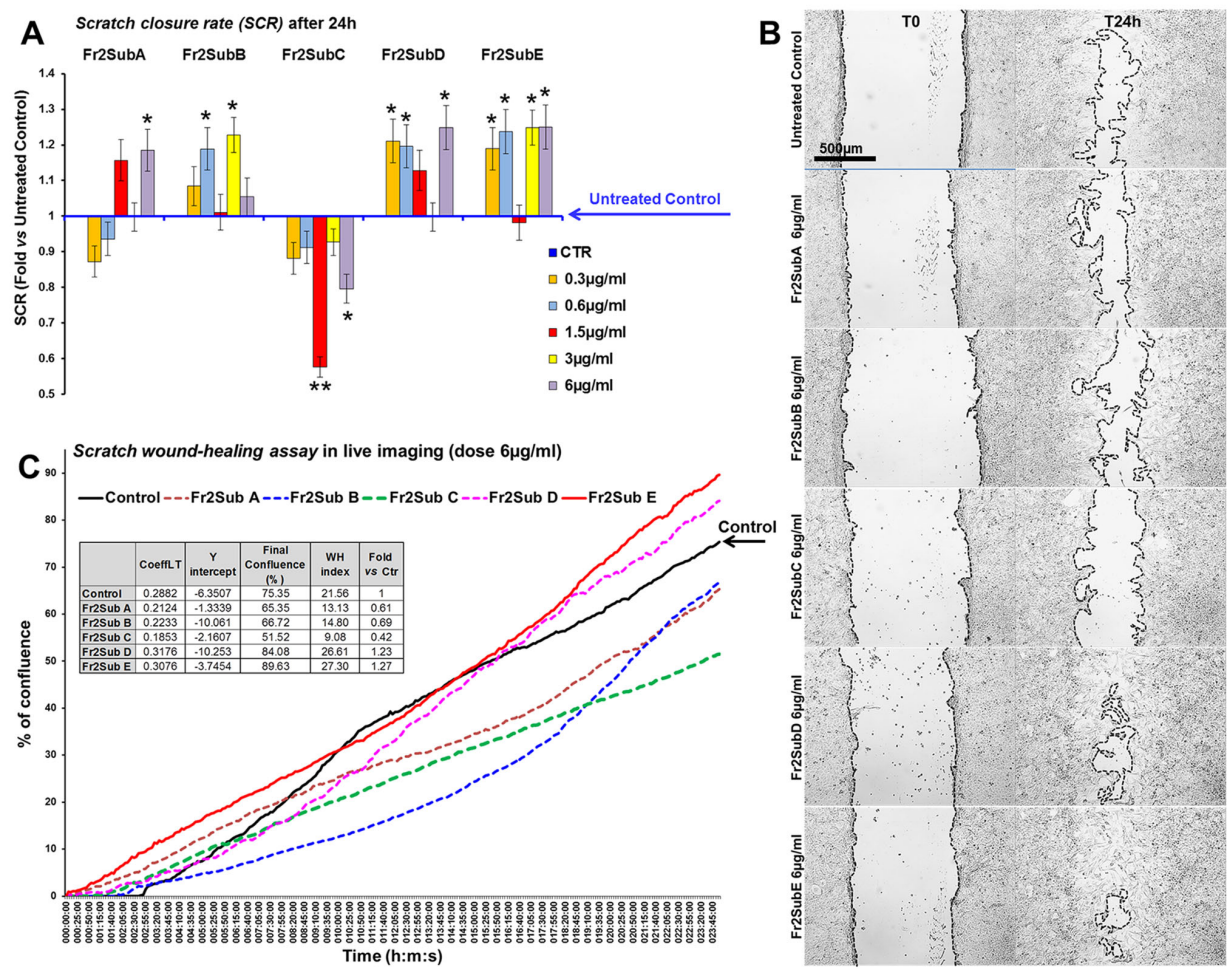
Effect of Fr2 sub-fractions A, B, C, D and E on scratch wound closure in 3T3 cell monolayer. **(A, B)** Scratch wound-healing assay performed, in static imaging modality, before (T0) and after 24 h (T24h) of treatment with 0.3, 0.6, 1.5, 3 and 6 µg/ml of the sub-fractions, compared to the untreated control. **(A)** Bar graph of scratch closure rates (SCR) at T24h, calculated as described in the method section. Significance *vs* untreated control (CTR): *p < 0.05; **p < 0.01; the mean ± SD; n = 3. **(B)** Phase contrast microscopy of 3T3 cells before (T0) and after 24 h (T24) of treatment with 6µg/ml of sub-fractions A, B, C, D and E, compared to the untreated control. Bar: 500µm. **(C)** Graph from the time-lapse imaging in bright field on living 3T3 cells. Cells were monitored for 24 h of culture in absence and in presence of 6 µg/ml of the Fr2 sub-fractions (the higher doses used in the static imaging modality), and the wound healing rate was automatically recorded as percentage of confluence in function of time. From the quantitative data of the wound healing dynamic, the wound healing index (WH) was calculated as describe in the method section (table in panel **C)**. CoeffLT = slope coefficient of linear trend-line equation (directly correlated to the speed of wound closure); *y* intercept = the point where the trend-line crosses the y axis (inversely correlated to the time at which the first migratory movement is recorded and, therefore, to the promptness of the effect).

Finally, the scratch wound-healing assay, performed by both static and time-lapse imaging (**Figure 8**) confirmed that the sub-fractions D and E possessed the higher ability in accelerating the wound closure compared to the other three fractions (the mean SCRs for all concentration tested of A, B, C, D and E sub-fractions were 82.3, 89.05, 65.5, 92.5 and 94.5, respectively) but the Fr2subE was lightly more effective than Fr2subD (**Figures 8B, C**), and acts in quite linear dose-dependent manner (**Figure 8A**). This strongly suggested that both Fr2subD and Fr2subE fractions contained the bioactive component but in the Fr2subE it was probably present at higher concentration and/or with a higher grade of purity than in the Fr2subD.

This hypothesis is confirmed by the chromatographic profiles of Fr2subD and Fr2subE (**Figure 6**): Fr2subE shows only one peak at 46 min while Fr2subD shows the same peak but an additional broad peak between 32 min and 42 min. Likely, the common peak is the compound responsible for the assessed biological activity and the broad peak represents the impurity that negatively influences the activity of Fr2subD.

### Identification of the Bioactive Compound from Fr2 Sub-Fraction E

An ESI-MS direct infusion in the positive ion mode of the bioactive Fr2 sub-fraction E revealed the presence of a dominant ion signal at m/z 429.4 [M+Na]^+^ (**Supplementary Figure 5**). ^1^H and ^13^C-NMR experiments (**Supplementary Figures 6** and **7**), in agreement with MS and MS^2^ data, allowed to unambiguously identify the isolated compound as nigracin (**Figure 1**).

NMR spectral data were in accordance with literature data (Sashidhara et al., 2013) while literature data on MS^2^ spectra are not available (**Supplementary Figure 5**).

### Isolation of the Bioactive Compound from DME

Once nigracin was identified in Fr2 sub-fraction E and fully characterized, a semi-preparative chromatography applied on the crude DME allowed faster isolation of the pure compound, as described in the Materials and Methods section. Therefore, we were able to calculate the relative content of nigracin in the dried stem bark of *D. klainei* as 0.022 % (w/w).

**Figure 9:**
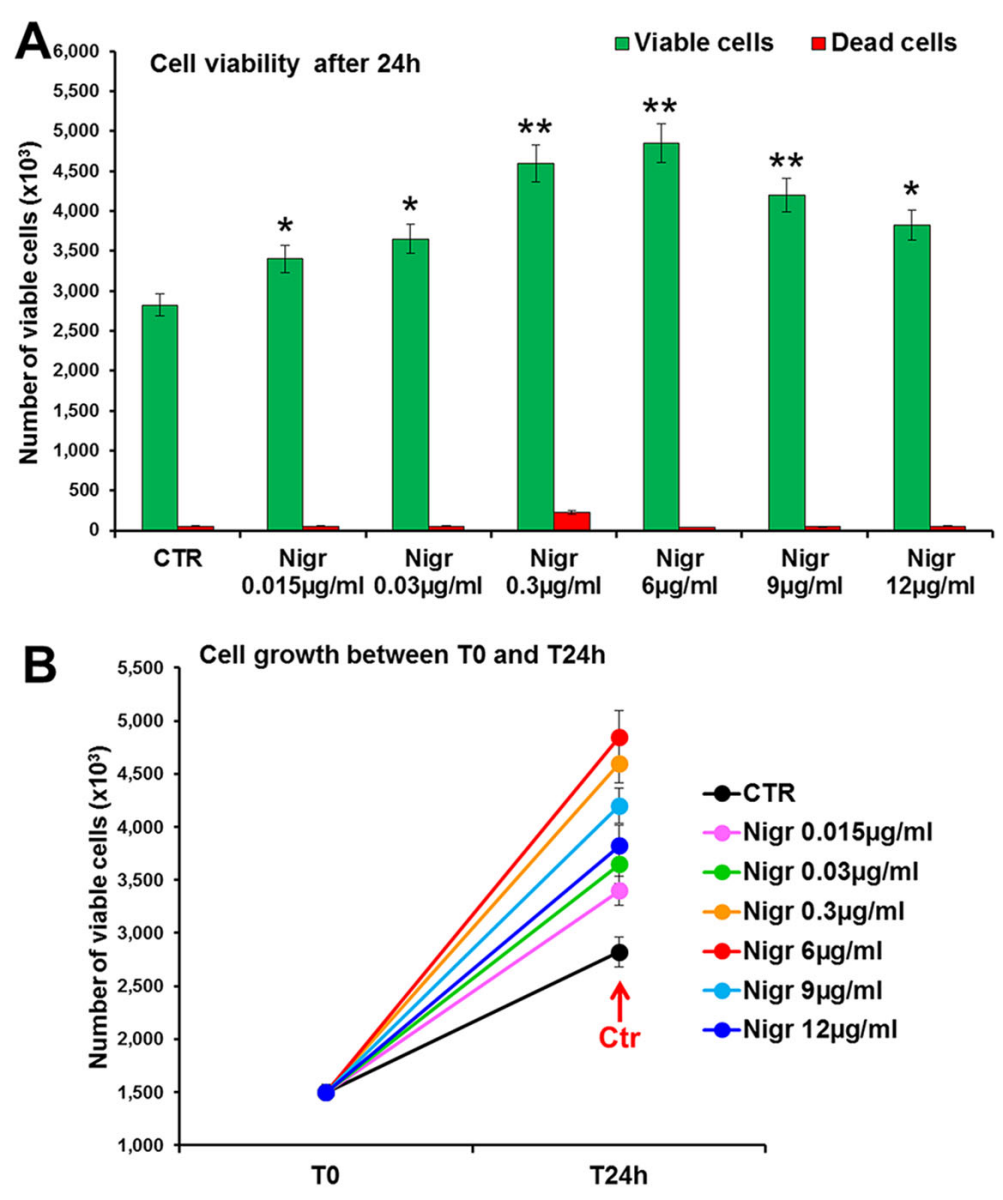
Effect of increasing concentrations of nigracin on 3T3 cell viability and growth. Cell viability (*top panel*) and growth (*bottom panel*), were evaluated by Trypan blue dye exclusion method, in untreated controls and cells treated for 24 h with 0.015, 0.03, 0.3, 6, 9 and 12 µg/ml of Nigracin. Significance *vs* untreated control (CTR): *p < 0.05; **p < 0.01; the mean ± SD; n = 3.

**Figure 10:**
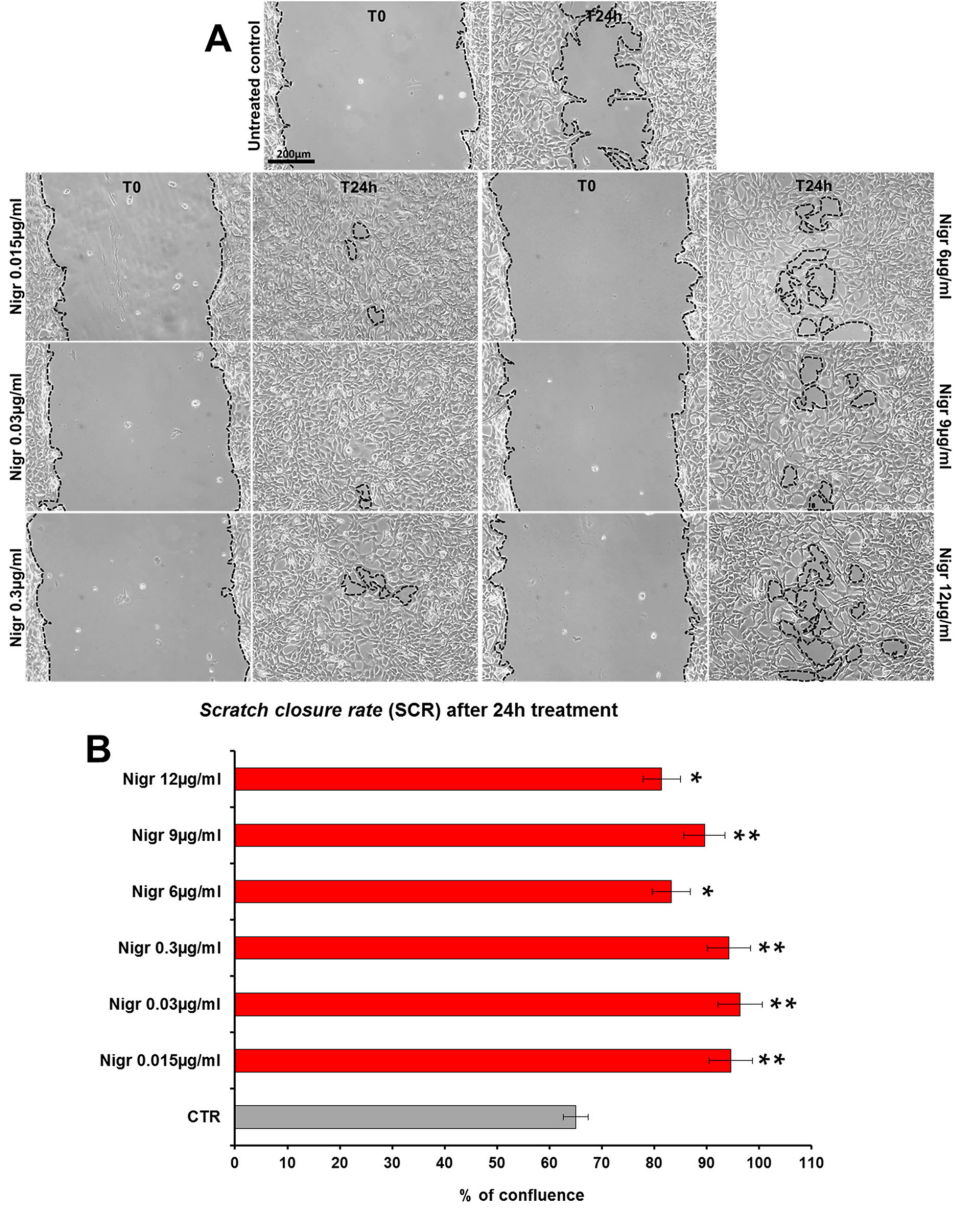
Effect of increasing concentrations of nigracin on scratch wound closure in fibroblast monolayer. 3T3 cells were treated with 0.015, 0.03, 0.3, 6, 9 and 12 µg/ml of nigracin and wound closure was analyzed after 24 h (T24h) of treatment by static imaging modality. **(A)** Representative images, by phase contrast microscopy, of 3T3 cells before (T0) and after 24 h (T24h) of treatment, compared to the untreated control. Bar: 200 µm. **(B)** Bar graphs of scratch closure rates (SCR) at T24h, calculated as described in the method section. Significance *vs* untreated control (CTR): *p < 0.05; **p < 0.01; the mean ± SD; n = 3.

**Figure 11:**
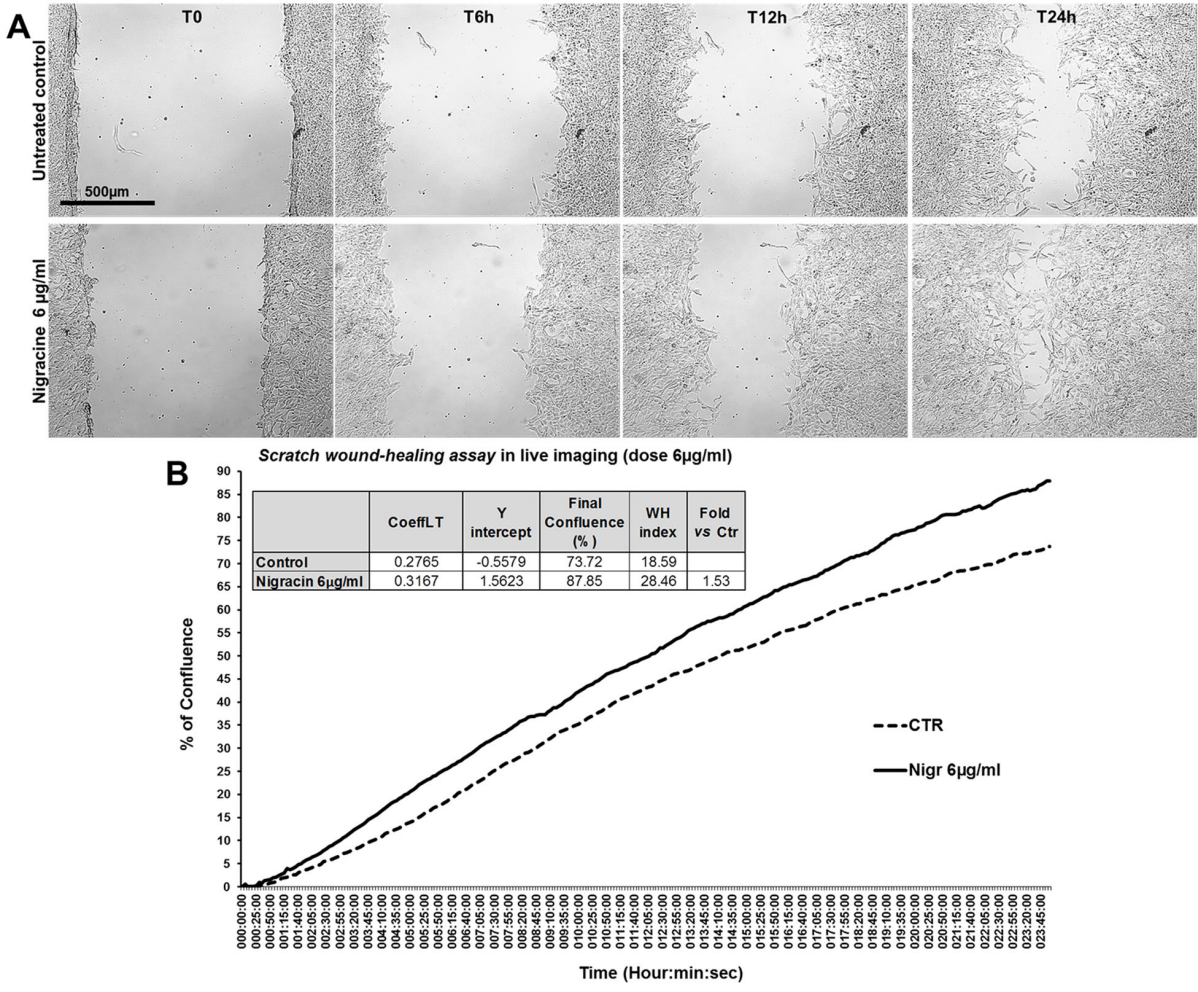
Effect of nigracin on the dynamic of scratch wound closure in fibroblast monolayer. **(A)** Phase contrast microscopy from time-lapse acquisition on 3T3 cells at T0 and after 6 h (T6h) and 24 h (T24) of treatment with 6µg/ml of nigracin, compared to the untreated control. Bar: 500µm. **(B)** Graph from the time-lapse imaging in bright field on living 3T3 cells. Cells were monitored for 24 h of culture in absence and in presence of 6 µg/ml of the compound, and the wound healing rate was automatically recorded as percentage of confluence in function of time. From the quantitative data of the wound healing dynamic, the wound healing index (WH) was calculated as describe in the method section (table in panel **B**).

The bioactivity of nigracin isolated from the crude extract was then tested on 3T3 cells, by analyzing the effects on cell viability and growth and on cell migration. The results obtained confirmed that nigracin was the active compound responsible for the tissue repair activity of DME extract and DME-derived fractions. Specifically, increasing concentrations of the compound (0.015, 0.03, 0.3, 6, 9 and 12 µg/ml) were able to stimulate fibroblast growth in a dose-dependent manner (reaching a plateau between 6 and 9 µg/ml), without any toxicity (**Figure 9** and **Supplementary Table 3**) and to significantly improve cell motility and wound closure, already at the lower dose used (0.015 µg/ml) (**Figure 10** and **Supplementary Table 3**). The scratch wound-healing assay, performed for 24 h by time-lapse imaging using the intermediate concentration of 6 µg/ml, confirmed that nigracin accelerated of 1.5-fold the dynamic of wound closure compared to the untreated control (**Figure 11** and **Supplementary Videos 1** and **2**).

**Figure 12:**
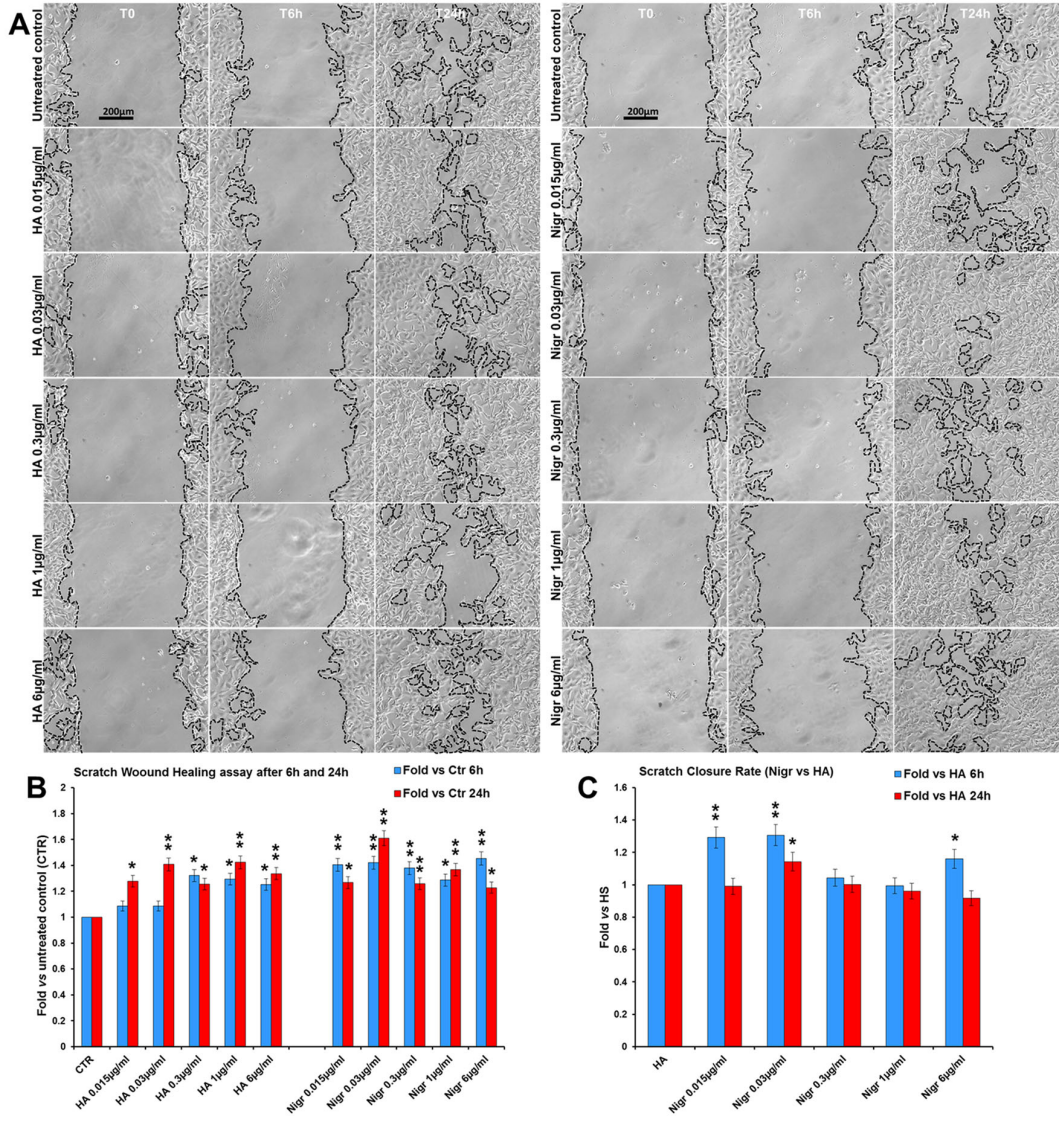
Comparative analysis of the effect of increasing concentrations of hyaluronic acid (HA; intermediate molecular weight: 500-700 kDa) and nigracin (Nigr) on scratch wound closure in fibroblast monolayer. 3T3 cells were treated with 0.015, 0.03, 0.3, 1 and 6 µg/ml of nigracin or HA and wound closure was analysed after 6 h (T6h) and 24 h (T24h) of treatment by static imaging modality. **(A)** Representative images, by phase contrast microscopy, of 3T3 cells before (T0) and after 6 h (T6h) and 24 h (T24h) of treatments, compared to the untreated control. Bar: 200 µm. **(B, C)** Bar graphs of scratch closure rates (SCR) at T6h and T24h, calculated as described in the method section, and reported as fold in nigracin and HA *vs* untreated control **(B)** and as fold in nigracin *vs* HA **(C)**. Significance *vs* untreated control (CTR) or *vs* HA: *p < 0.05; **p < 0.01; the mean ± SD; n = 3.

Finally, in order to assess the efficacy of nigracin *vs* a positive control of wound healing stimulation, we performed a comparative analysis of the effect on scratch wound closure of increasing concentrations of nigracin and hyaluronic acid (HA; intermediate molecular weight: 500-700 kDa), the extracellular matrix component that is known to be extensively involved in all phases of wound healing and that is currently used in biomedical applications for stimulating skin repair (Frenkel, 2014; D’Agostino et al., 2015). To this purpose, 3T3 cells were treated with 0.015, 0.03, 0.3, 1 and 6 µg/ml of nigracin or HA and wound closure was analyzed after 6 h (T6h) and 24 h (T24h) of treatment by static imaging modality. The results obtained showed that nigracin improved the scratch wound closure with an efficacy comparable to that of the positive control HA (**Figures 12A, B**). In addition, the nigracin-induced stimulation seems to be more prompt than that induced by HA, as demonstrated by the about 1.3-fold higher SCR recorded at T6h for nigracin vs HA (**Figure 12C**). Moreover, cell viability assay showed that nigracin was even less toxic than HA (**Supplementary Figure 8**).

## Discussion

Herbal preparations have been used for centuries as the major source of health care for the world’s population. In the last years, with advances of technologies that allow the screening of natural products in high-throughput assays, the interest in plant-derived drugs has progressively increased and a “New Golden Age” for the drug discovery of natural-derived products is emerging (Harvey et al., 2015; Shen, 2015).

The stem bark of *Drypetes klainei* is traditionally used, by Baka people in Cameroon, as water macerated extract in the wound healing process and for the treatment of burns. We have previously validated the traditional use of this herbal preparation in the treatment of skin lesions, demonstrating that both water (WE) and defatted methanol (DME) extracts are able to accelerate scratch wound closure in fibroblast cultures (Brusotti et al., 2015). In this work, starting from those published results, we carried out a bioassay-guided fractionation of the most active DME extract, with the aim of identifying and isolating the bioactive compound/s responsible for the wound repair activity. The DME extract was analyzed by RP-HPLC and purified by flash chromatography giving rise to three fractions. The fraction Fr2, that exhibited the higher activity on wound closure, was re-fractioned into sub-fractions, which were subjected to the bioassays. Among the five sub-fractions obtained from the Fr2 (Fr2subA, B, C, D, and E), the Fr2subE resulted the most active in affecting cell viability and growth as well as on cell migration assessed by scratch wound assays. The Fr2subE, containing only one dominant component, as shown in the chromatographic profile, was directly analyzed by direct infusion ESI-MS. The m/z 429.4 [M+Na]^+^ matched with different molecular formulae, but the fragmentation pattern suggested C_20_H_22_O_9_ as the most probable one. This brute formula has been exploited to perform a Chemical Abstract search, which afforded about 250 molecular structures. By refining the search based on the NMR data, in particular focusing the attention on substitution pattern of the aromatic rings, we finally identified three plausible chemical structures. One of them corresponded to Pilorubrosin, a glycoside firstly isolated from the leaves of *Protea Rubropilosa* Beard (Perold et al., 1973), while the others were identified as benzoylated gentisyl alcohol glucosides: Iotoside A (Chai et al., 2007) and nigracin (Sashidhara et al., 2013), differing for the position of the benzoyl group. The NMR spectra in our hands are completely in agreement with those reported for nigracin.

Nigracin, firstly isolated and characterized from bark and leaves of *Populus nigra* in 1967 (Thieme and Benecke, 1967), was never found before in the genus *Drypetes* nor a wound healing activity has been demonstrated for this molecule.

Here we demonstrated that nigracin significantly stimulates fibroblast growth and improves cell motility and wound closure of fibroblast monolayer in a dose-dependent manner, without any toxicity at the concentrations tested (up to 12 µg/ml) and is still active at very low doses (0.015 µg/ml). This makes the molecule particularly attractive as a possible candidate for developing new therapeutic options for wound care, alternative and perhaps more efficient than those currently available. In this regard, the results obtained by comparing the effects on scratch closure of nigracin and hyaluronic acid (**Figure 12**), strongly suggest that nigracin could be more efficient in promoting wound repair than the hyaluronic acid, possibly acting by a different mechanism that might result in a more prompt therapeutic response. Additional studies will be carried out to confirm the nigracin efficacy in *3D* cultures and/or in an *in vivo* experimental model and to clarify the mechanism of action through which this molecule stimulates wound closure. Moreover, since the isolation of the bioactive nigracin from the DME extract involves a number of disadvantages, including the time consuming, the cost of the methodologies used, the limited yield and difficulties in getting the *D. klainei* stem barks, the development of a method for obtaining the synthetic nigracin should be one of the main goal of upcoming studies.

As a conclusive remark, we would like to emphasize that the methodological approach and the bioassays used in this work have allowed us not only to demonstrate that nigracin is the bioactive compound responsible for the wound repair activity exhibited by the *D. klainei* stem bark extract but also to identify other sub-fractions from the Fr2, that have bioactivities different and in some case opposite to those of Fr2SubE and that would be worthy of deepening in future studies.

## Data Availability Statement

The datasets for this article are not publicly available because they are still unpublished. Requests to access the datasets should be directed to AS, annalucia.serafino@ift.cnr.it; GB, gloria.brusotti@unipv.it.

## Author Contributions

GS contributed to design of the study, helped to draft the manuscript, and revised it critically. MC performed the chromatographic analyses, helped to draft the manuscript, and revised it critically. FA, DG, and MZ performed the experiments of cell viability and growth and cell cycle and wound healing. GN and PP helped to draft the manuscript and revised it critically. ST performed the mass spectrometry analyses. MS carried out the NMR analyses. EC and GB designed the study on chemical analysis and structural characterization, helped to draft the manuscript, and revised it critically. AS designed the bio-assay studies, supervised all experiments, analyzed the data, carried out the statistical analysis, and drafted the manuscript. All authors read and approved the final manuscript.

## Funding

This work was partially supported by IFT-CNR (National Research Council of Italy) (Project DSB.AD007.088 to AS).

## Conflict of Interest

The authors declare that the research was conducted in the absence of any commercial or financial relationships that could be construed as a potential conflict of interest.

## Supplementary Materials

Supplementary Video 1 Time-lapse imaging of wound healing in murine fibroblast cell line 3T3, monitored for 24 h of culture in absence of nigracin (untreated control), by acquiring 1 frame/5 min.

Supplementary Video 2 Time-lapse imaging of wound healing in murine fibroblast cell line 3T3, monitored for 24 h of culture in presence of 6 µg/ml of nigracin, by acquiring 1 frame/5 min.
