# Supplementary material for: Bioassay-Guided Isolation of Nigracin, Responsible for the Tissue Repair Properties of Drypetes Klainei Stem Bark
Source: Front Pharmacol. 2020 Jan 23;10:1541. doi: 10.3389/fphar.2019.01541 (PMC6989535; doi:10.3389/fphar.2019.01541)
Supplement: Supplementary file 1 [file DataSheet_1.pdf]

SUPPLEMENTARY FIGURES

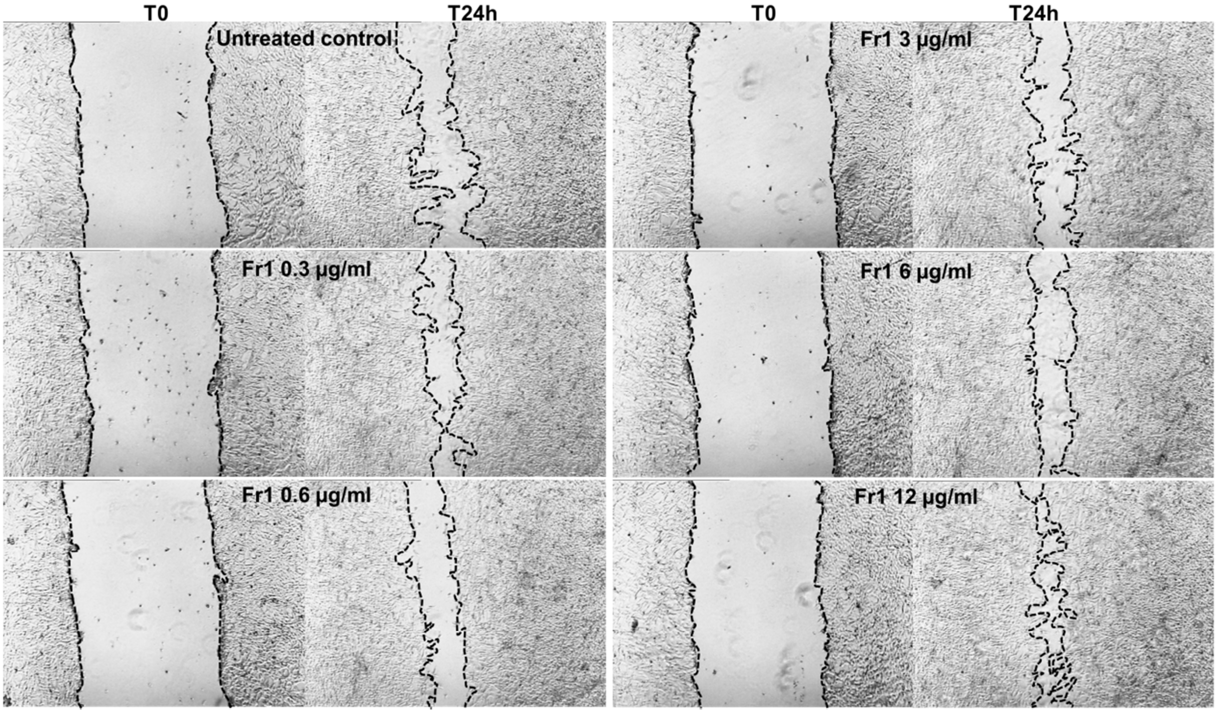

Supplementary Figure 1. Scratch wound closure (static imaging modality) in 3T3 cell monolayer. Phase contrast microscopy of 3T3 cells before (T0) and after 24 h (T24) of treatment with 0.3, 0.6, 3, 6 and 12µg/ml of Fr1, compared to the untreated control.

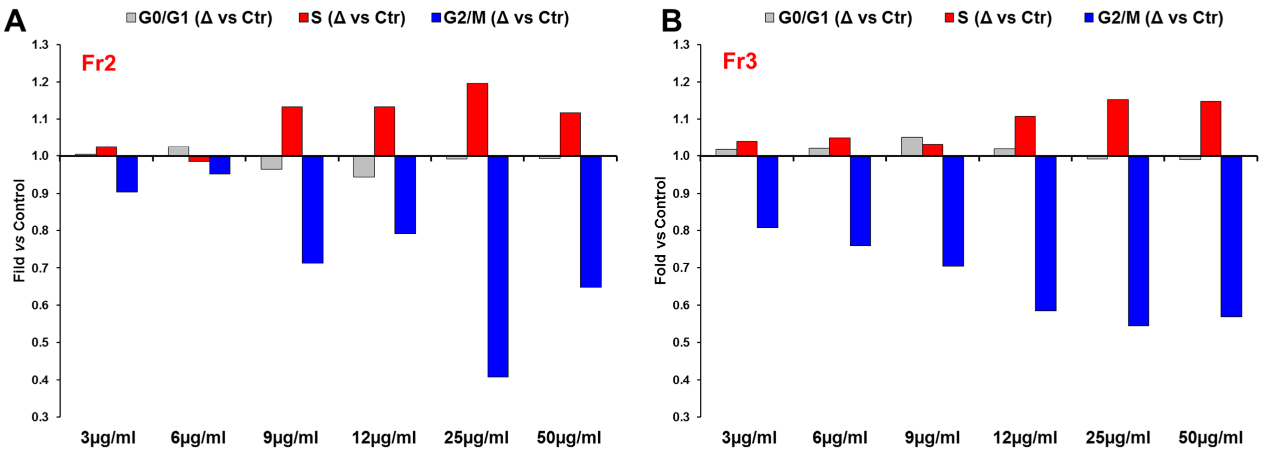

Supplementary Figure 2. Comparative analysis of the effect of increasing concentrations of Fr2 and Fr3 fractions on cell cycle of murine fibroblasts on cell cycle. Cytofluorimetric analysis was performed on 3T3 cells after 24 h of treatment with 3, 6, 9, 12, 25, 50 µg/ml of Fr2 (**A**) and Fr3 (**B**), compared to the untreated controls (represented in the bar graphs as *y* axis=1). Results a reported as fold *vs* untreated control for each cell cycle phase.

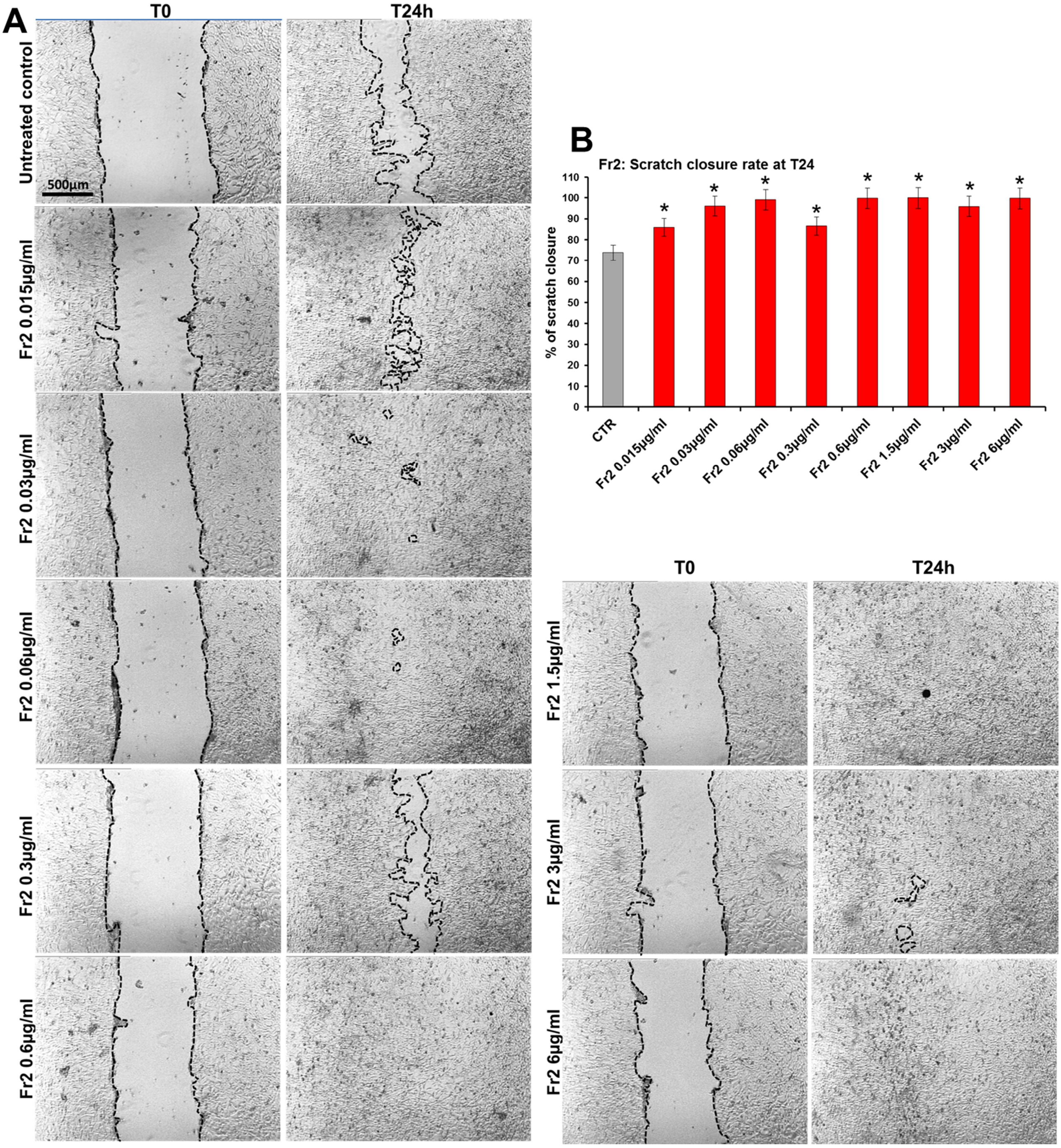

Supplementary Figure 3. **Effect of increasing concentrations of Fr2 fraction on scratch wound closure in 3T3 cell monolayer.** 3T3 cells were treated with 0.015, 0.03, 0.06, 0.3, 0.6, 1.5, 3,and 6 µg/ml of Fr2 and wound closure was analysed after 24 h (T24h) of treatment by static imaging modality. **A)** Representative images, by phase contrast microscopy, of 3T3 cells before (T0) and after 24 h (T24h) of treatment, compared to the untreated control. Bar: 500 µm. **B)** Bar graphs of scratch closure rates (SCR) at T24h, calculated as described in the method section. Significance *vs* untreated control (CTR): *$p < 0.05$; the mean ± SD; n = 3

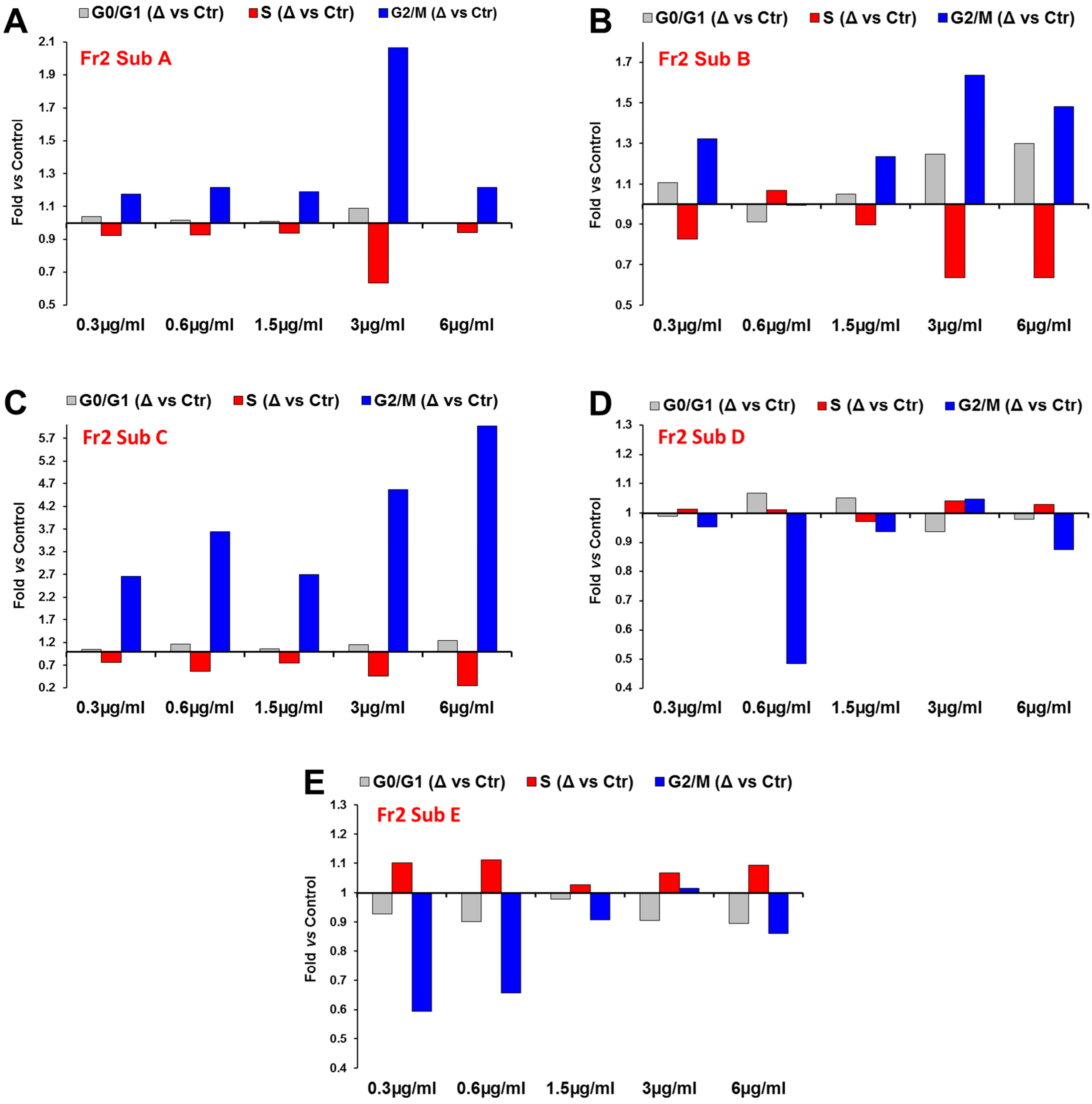

Supplementary Figure 4. Comparative analysis of the effect of increasing concentrations of Fr2subA, B, C, D and E fractions on cell cycle of murine fibroblasts. Cytofluorimetric analysis was performed on 3T3 cells after 24 h of treatment with 3, 6, 9, 12, 25, 50 µg/ml of Fr2subA (**A**), Fr2subB (**B**), Fr2subC (**C**), Fr2subD (**D**) and Fr2subE (**E**), compared to the untreated controls (represented in the bar graphs as *y* axis=1). Results are reported as fold *vs* untreated control for each cell cycle phase.

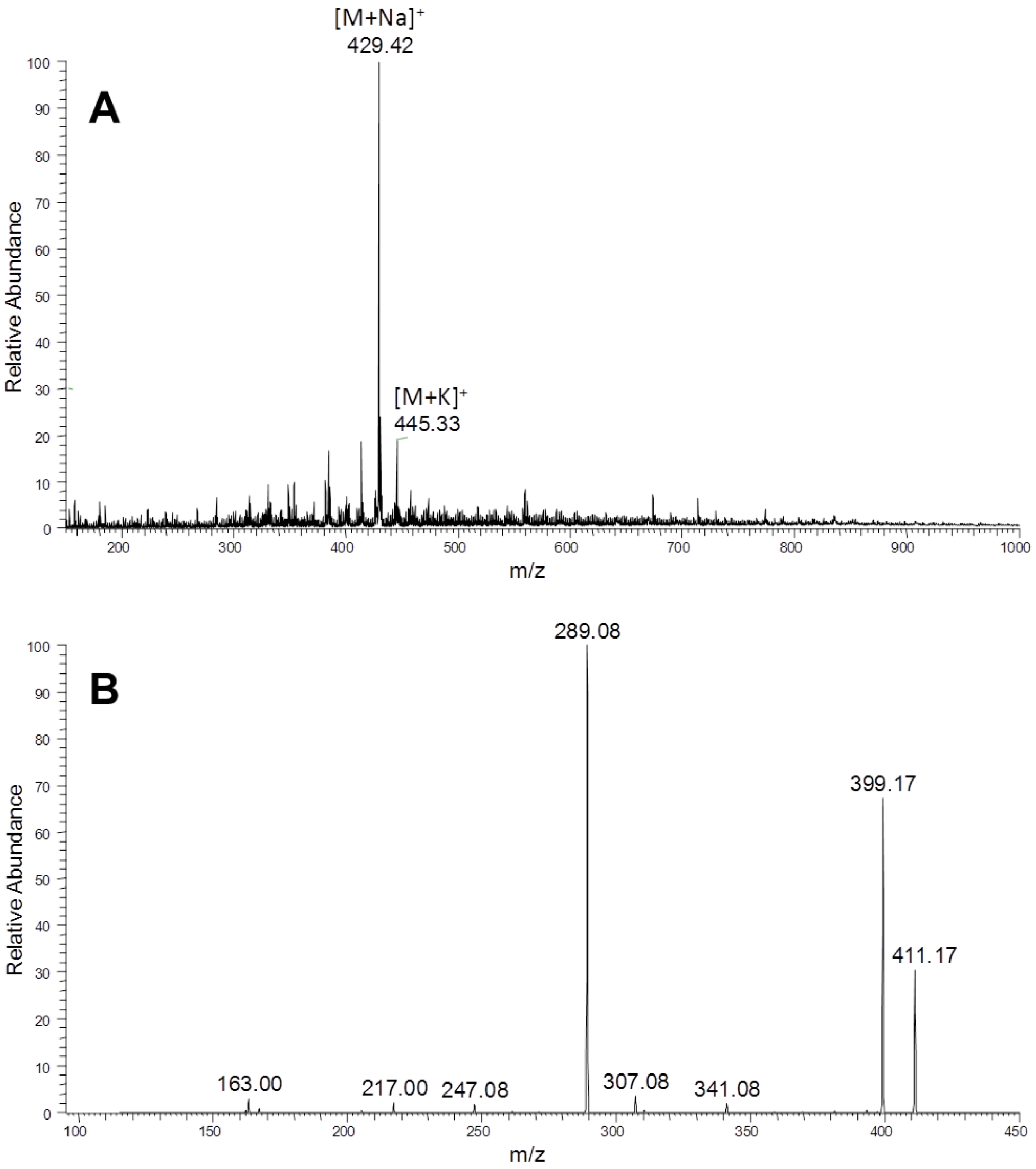

Supplementary Figure 5. **Spectrometric analyses. A**) Full scan MS spectrum and **B**) MS/MS spectrum for m/z 429 obtained by direct infusion ESI-MS analysis of Fr2 sub-fraction E

Nigracin or Poliothrysoside

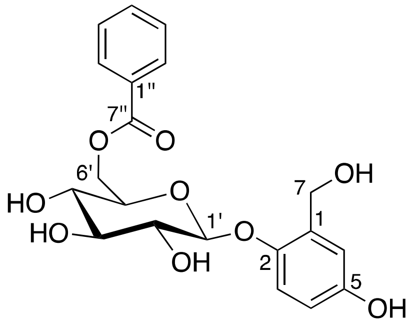

^{1}H NMR (CD_3OD, 400 MHz) δ 3.43–3.54 (m, 3H, H2'–H4'), 3.73 (m, 1H, H5'), 4.45 (dd, *J* = 7.3, 11.8 Hz, 1H, H6'), 4.50 (d, *J* = 13.2 Hz, 1H, H7), 4.71–4.77 (m, 2H, H1' + H6'), 4.73 (d, *J* = 13.2 Hz, 1H, H7), 6.48 (dd, *J* = 3.0, 8.7 Hz, 1H, H4), 6.79 (d, *J* = 3.0 Hz, 1H, H6), 7.03 (d, *J* = 8.7 Hz, 1H, H3), 7.53 (t, *J* = 7.8 Hz, 2H, H3'' + H5''), 7.66 (t, *J* = 7.4 Hz, 1H, H4''), 8.05 (d, *J* = 7.8 Hz, 2H, H2'' + H6'')

^{13}C NMR (CD_3OD, 100 MHz) δ 61.3 (t), 65.7 (t), 72.3, 75.4, 75.9, 78.3, 104.8, 115.8, 116.5, 119.8, 129.9, 131.0, 131.6 (s), 134.3 (s), 134.7, 150.2 (s), 154.5 (s), 168.1 (s)

Supplementary Figure 6. ^{1}H NMR and ^{13}C NMR data of Nigracin.

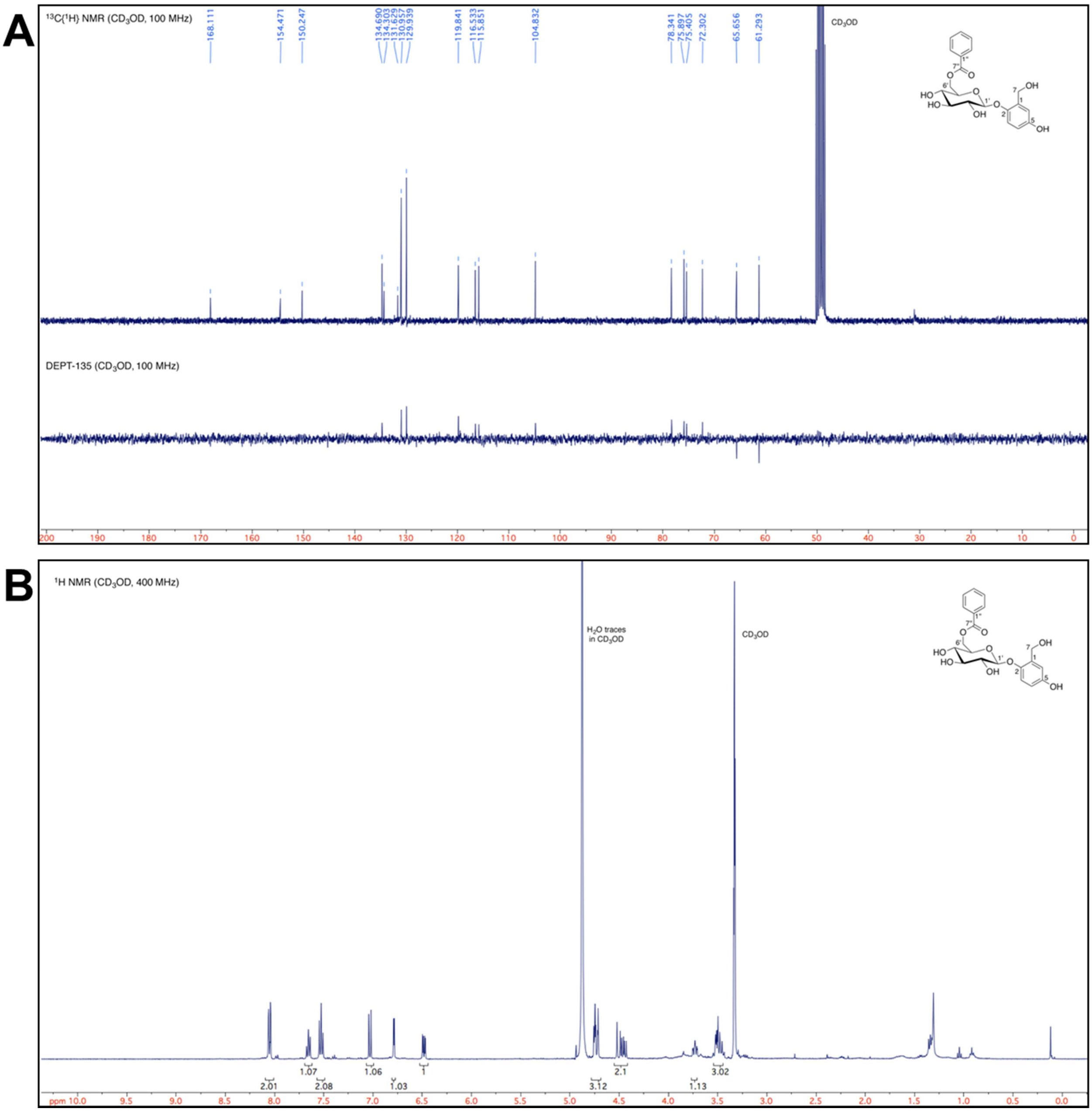

Supplementary Figure 7. ^{13}C NMR (A) and ^{1}H NMR (B) spectra of Nigracin.

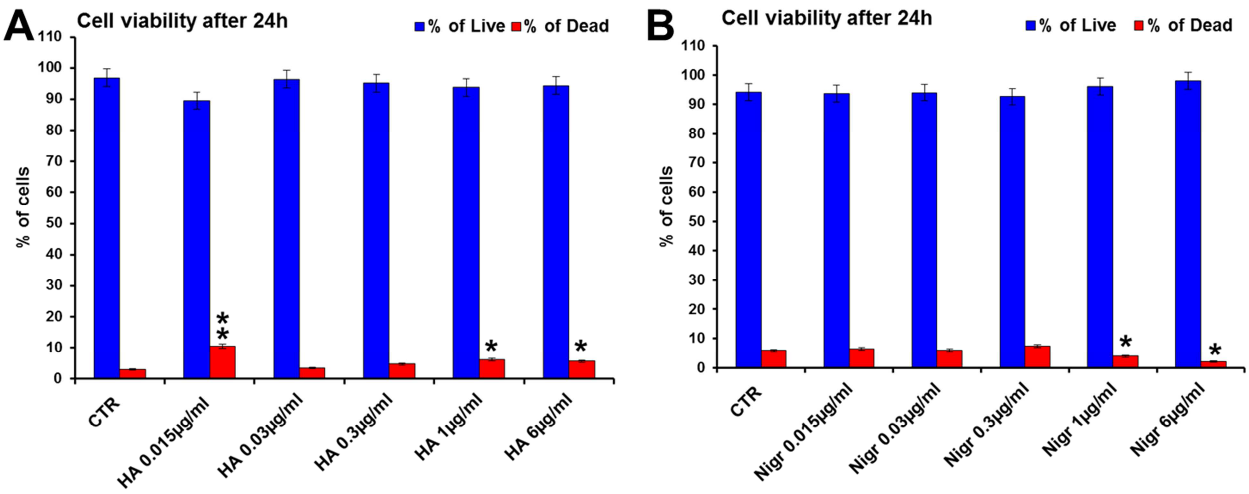

Supplementary Figure 8. Comparative analysis of the effect of increasing concentrations of hyaluronic acid (HA; intermediate molecular weight: 500-700 kDa) and nigracin (Nigr) on 3T3 cell viability. Cell viability was evaluated by Trypan blue dye exclusion method, in untreated controls and cells treated for 24 h with 0.015, 0.03, 0.3, 1 and 6 µg/ml of HA (**A**) and Nigr (**B**). Significance vs untreated control (CTR): *p < 0.05; **p < 0.01; the mean ± SD; n = 3
