# Supplementary material for: Bioassay-Guided Isolation of Nigracin, Responsible for the Tissue Repair Properties of Drypetes Klainei Stem Bark
Source: Front Pharmacol. 2020 Jan 23;10:1541. doi: 10.3389/fphar.2019.01541 (PMC6989535; doi:10.3389/fphar.2019.01541)
Supplement: Supplementary file 2 [file DataSheet_2.pdf]

SUPPLEMENTARY TABLES

Supplementary Table 1. Resuming table of the effects of Fr2 and Fr3 fractions on scratch wound closure

	Scratch wound healing activity (Fold *vs* untreated control)			
	Fr2 fraction		Fr3 fraction	
	T6h	T24h	T6h	T24h
3 µg/ml	**1.78**	**1.26**	0.98	**1.18**
6 µg/ml	**1.46**	**1.25**	0.88	1.10
9 µg/ml	**1.64**	**1.25**	1.12	1.08
12 µg/ml	0.90	**1.27**	**1.40**	1.08
25 µg/ml	**1.56**	**1.27**	**2.12**	1.00
50 µg/ml	**1.37**	**1.27**	0.96	1.10

The significant values are highlighted in bold

Supplementary Table 2. Resuming table of the effects of sub-fractions from Fr2 on cell death after 24h of treatment

	Percent of dead cells*				
	**Fr2subA**	**Fr2subB**	**Fr2subC**	**Fr2subD**	**Fr2subE**
CTR	3.16	3.16	3.16	3.16	3.16
0.3 µg/ml	5.41	1.41	3.51	2.78	5.8
0.6 µg/ml	13.04	1.32	1.52	4.76	5.48
1.5 µg/ml	10.71	2.78	8.33	3.41	6.25
3 µg/ml	2.44	1.23	2.65	5.21	9.09
6 µg/ml	16.67	2.44	4.55	4.55	10.11

*Mean values; n=3

Supplementary Table 3. Resuming table of the effects of nigracin on cell death, growth and migration after 24h of treatment

	Nigracin effects* (Fold *vs* untreated control)		
	**% of dead cells**	**Cell growth**	**SCR**
CTR	1	1	1
0.015 µg/ml	0.86	1.2	1.45
0.03 µg/ml	0.83	1.3	1.48
0.3 µg/ml	2.67	1.6	1.45
6 µg/ml	0.46	1.7	1.28
9 µg/ml	0.61	1.5	1.38
12 µg/ml	0.75	1.4	1.25

*Mean values; n=3
